# A Structured Narrative Literature Review of the Broader Value of Adult Immunisation Programmes

**DOI:** 10.3390/vaccines12080852

**Published:** 2024-07-29

**Authors:** Hania El Banhawi, Eleanor Bell, Margherita Neri, Simon Brassel, Sulayman Chowdhury, Lotte Steuten

**Affiliations:** Office of Health Economics, London SE1 2HD, UK; helbanhawi@ohe.org (H.E.B.); eleanor.bell@ukhsa.gov.uk (E.B.); mneri@ohe.org (M.N.); sbrassel@ohe.org (S.B.); schowdhury@ohe.org (S.C.)

**Keywords:** health technology assessment, vaccine evaluation, vaccine value, public health, healthy ageing, societal impact

## Abstract

Vaccine-preventable diseases continue to generate a substantial burden on health, healthcare systems, and societies, which is projected to increase with population ageing. There is a need to better understand the full value of adult immunisation programmes corresponding to the broader value of vaccine frameworks that are recommended for evidence-based decision-making. This review aims to summarise and map evidence for the value of selected adult immunisation programmes (seasonal influenza, pneumococcal disease, RSV, and HZ) in ten diverse countries. We conducted a structured literature review of evidence published from 2017 to 2023. An existing framework was used to structure the assessment, developing matrices demonstrating the elements of value evidenced for each vaccine and country of focus. Our analysis showed substantial evidence base on the value of adult immunisation programmes, but the availability of evidence varied by value element and by vaccine. The impact on the quality of life of the vaccinated individual was the most evidenced value element. Mortality benefits for vaccinated individuals and cost-offsets to healthcare systems were also well-evidenced. The availability of evidence for ‘broader’ societal value elements (such as transmission value, carer productivity and impact on social equity, and antimicrobial resistance prevention) varied. No evidence was identified relating to the broader value elements of macroeconomic effects, value to other interventions, or effects on the quality of life of caregivers. Robust evidence exists to show that adult immunisation programmes generate substantial value for population health and health systems, yet some elements of broader value remain underrepresented in the academic literature. Without such evidence, the full value of immunisation programmes is underestimated, risking suboptimal policy decisions.

## 1. Introduction

Vaccines are amongst the most effective interventions in medical history [1], having eliminated or even eradicated diseases like yellow fever, diphtheria, cholera, polio, measles, and rubella [2]. Today, however, other vaccine-preventable diseases generate a substantial burden on health, healthcare systems, and societies around the world. Over 51 million deaths are expected to be averted due to vaccination against 14 pathogens between 2021 to 2030, which translates to about 5.2 million deaths averted per year [3]. The prioritisation of preventive healthcare, including vaccines, is increasingly recognised as essential for supporting healthcare systems and societies to respond to these challenges [4]. Yet, despite significant progress in ensuring global access to childhood immunisation programmes since the establishment of dedicated global initiatives and immunization goals, progress on vaccinating adults and older adults has been limited [5,6,7]. 

Vaccine-preventable diseases continue to produce substantial disease and mortality burdens in adult populations worldwide. For example, disaggregated data focusing on vaccine-preventable diseases estimated that one in five communicable-disease deaths among adults aged 60 and older were attributable to three priority vaccine-preventable diseases (influenza lower respiratory tract infections, pneumococcal pneumonia and meningitis, and herpes zoster) [8]. Despite this, access to vaccinations varies widely, with many countries excluding adult vaccinations from routine schedules [9,10]. For example, reported influenza vaccination coverage rates among older adults vary significantly, with rates as low as 9.2% in Saudi Arabia and as high as 81.9% in South Korea [11].

The value of adult immunisation programmes continues to be under-recognised in the academic literature and decision-making frameworks globally [12,13,14]. Beyond preventing illness, vaccines can generate substantial ‘externalities’ (i.e., indirect spillover effects on third parties) [15]. The Broader Value of Vaccines framework by Bell et al. [16] categorises and conceptualises the distinct elements of vaccines’ value based on a synthesis of the literature. The framework distinguishes between ‘narrow’ effects of vaccines, concerning the impact of vaccines on the health of individuals and the health and economic effects on the health system, and ‘broader’ effects of vaccines, which include the effects of vaccines beyond those vaccinated, as well as various socio-economic effects outside of the health system [16]. It has been widely debated in the literature that policy and decisionmakers typically only consider narrow health effects and economic effects within the health system, but not necessarily effects external to these [12,16,17,18]. This under-recognition of the value of adult vaccines may lead to suboptimal decision-making by policymakers and formulary decision-makers at the national or subnational level. 

Aligning with calls to integrate recommended immunizations throughout the life course [19] and to expand on the narrow frameworks traditionally used to evaluate the value of immunization [17], this study aims to synthesise and categorise evidence from ten diverse countries on the value of various adult immunisation programmes for health, healthcare systems, and societies according to the Broader Value of Vaccines framework, and to identify under-recognised value elements of these adult immunisation programmes. 

## 2. Materials and Methods

This targeted literature review sought to identify evidence on the value of four adult immunisation programmes (seasonal influenza, pneumococcal disease, respiratory syncytial virus (RSV), and herpes zoster (HZ)) which prevent infections due to seasonal influenza (flu), streptococcus pneumonia, RSV, and varicella zoster virus (VZV), respectively. The four adult vaccines were selected given their substantial and established disease burden now and in future projections [8]. The selection allows us to differentiate between those vaccines that mainly generate value by preventing mortality (PD, influenza, and RSV) and those that mainly generate value by preventing morbidity (HZ). 

We focused on ten countries, carefully selected to represent a diversity of immunisation schedules, healthcare systems, geographies, demographic contexts, and vaccine schedules: Australia, Brazil, France, Germany, Italy, Japan, Poland, South Africa, Thailand, and the United States of America. For a breakdown of the immunisation schedules of the selected countries at the time of analysis, see Table S1 in the Supplementary Materials. 

The narrative synthesis looks beyond the traditional criteria used to estimate the cost-effectiveness of drugs, which typically focus on ‘narrow’ health and healthcare system effects. We focus on evidence within three key value domains: value for population health; value for healthcare systems; and value for society [14,16]. Following Cafiero-Fonseca et al. and Bell et al. [12,16], we assessed the strength of the evidence base for the value of adult immunisation programmes by reviewing the papers identified in the search to determine which elements of value have been evidenced for each vaccine and country of focus. The Broader Value of Vaccines framework by Bell et al. [16] was used to structure the assessment. The definition and characterisation (narrow/broad) of each of the eleven value elements of the framework is explained in Table 1. According to the framework, narrow value elements include the impact of vaccines on the health of vaccinated individuals and on economic effects within the health system. Broad value elements include the effects of vaccines beyond those vaccinated, as well as various socio-economic effects outside of the health system (e.g., productivity). We developed evidence matrices to demonstrate evidence availability for the value of adult immunisation as characterised by the framework. We assessed evidence availability per value element for each immunisation programme, overall and for each country. We remained vaccine-agnostic within each disease area, and excluded evidence relating solely to the burden of disease. 

The study design was a targeted literature review which employed the following inclusion criteria: quantitative and qualitative analyses, published from 1 January 2017 to 31 June 2023, full texts available, published in the English language, and studying adult human populations aged 18 and above. We excluded papers with only abstracts available, as well as case series, case reports, editorials or expert opinions. The initial search was restricted to evidence published since 2017 to prioritise the more recent literature showcasing the breadth of effects and given that very few studies published prior to 2017 considered effects beyond health benefits to the vaccinated individual and healthcare systems, such as population-level health benefits, productivity benefits, and other broader societal benefits [12]. Further, we reviewed the reference lists in the articles identified by the database search to capture additional relevant studies, including those that fell outside the time range, but captured broader effects and for which no other or more recent data were available. 

Two reviewers (H.B. and E.B.) independently screened the literature and extracted the following data: study type, author, study location, time frame of data collection, population considered, and study findings. We conducted the search in PubMed. The search strategy and search terms used are shown in Appendix A. We included evidence from a broad range of study types considering the breadth of the topic, as well as geographical differences, for example, in data availability and analytical capacity. To mitigate the risk of bias, we prioritised evidence from systematic reviews and excluded evidence from the lowest tiers of the evidence hierarchy (case series, case reports, editorials, or expert opinions) [21]. 

All results considering the relevant value elements, including those reporting null findings, were included in the matrices. Studies highlighted in the narrative synthesis on the value of vaccination were statistically significant with a confidence level of 95% or higher, unless otherwise stated.

## 3. Results

### 3.1. Assessment of the Evidence Base for the Value of Adult Immunisation Programmes

We found 159 papers that fit our search criteria, 95 of which met the inclusion criteria for the value framework matrices for our countries and vaccines of focus. These are listed in Table S2 in the Supplementary Materials. Table 2 displays the matrix with the percentage of sample countries for which relevant evidence was identified on each value element by immunisation programme. Matrices showing results by country are presented in Appendix B. 

The results showed that evidence availability for all vaccines was greatest for the ‘narrow’ benefits: quality of life and mortality benefits to vaccinated individuals and cost-offsets to healthcare systems. Across vaccines, evidence of these value elements was identified in each of the ten countries of focus. For each vaccine, effects on the quality of life of vaccinated individuals was the value element most consistently evidenced across countries (The definition of this value element in the Broader Value of Vaccines framework is the value of effects on the physical, mental, emotional, and social functioning of vaccinated individuals, and we include any outcomes relating to infections, morbidity and health-related quality of life within this definition).

A substantial evidence base exists on the value of vaccination for some ‘broader’ value elements. Productivity value was considered in at least one country for every vaccine except for RSV, although we identified evidence of the productivity burden associated with RSV [22]. Evidence for transmission value exists for all relevant vaccines (i.e., influenza, pneumococcal, and RSV, as there is limited transmissibility of VZV between adults).

There is a paucity of evidence relating to other ‘broader’ value elements. Evidence for effects on the productivity of carers exists in a small proportion of the countries, for all vaccines except RSV. For effects on social equity, there is evidence relating to one of the included vaccines (pneumococcal) in one country (the United States). For effects on antimicrobial resistance, there is evidence relating to one of the included vaccines (influenza) in one country (Australia). No evidence was identified relating to macroeconomic effects, value to other interventions, or effects on the quality of life of caregivers in any country in the sample. 

The value of influenza and pneumococcal vaccination was the most comprehensively evidenced, with evidence for seven value elements identified for each vaccine. Evidence of five value elements was identified for HZ vaccination and of four value elements for RSV vaccination. 

The level of evidence availability differed across countries. The most comprehensive evidence was available in the United States, where evidence of seven of the eleven value elements was identified. The least comprehensive evidence was available in Poland and Thailand, where evidence of only two and three elements, respectively, was identified. For country-specific availability of evidence, see the full matrices presented in Appendix B. 

It is important to note that consideration of a value element may be through outcomes reflecting only partial value. For example, whilst effects on patient productivity were considered in the majority of countries, this was often measured solely in terms of absenteeism from formal workplaces, excluding effects on presenteeism and the value of informal market activity (for example, working in the informal economy) and informal non-market activity (for example, caregiving by unemployed or retired adults).

### 3.2. Value of Adult Immunisation Programmes for Population Health

#### 3.2.1. Value of Adult Immunisation Programmes for Vaccinated Populations

There is ample evidence (see Table 3) demonstrating the health value of adult immunisation programmes with respect to the prevention of disease, disease sequelae with major health consequences, and mortality in older adults (most commonly defined as adults aged 65 and older, but sometimes from the age of 50 and adults with risk factors).

**Table 3:** Findings on the value of adult vaccination programmes in older adults and adults with comorbidities.

Health Value	Study Type	Geography	Time Frame	Population	Findings	Reference
Influenza						
Disease prevention	Systematic review and meta-analysis	Global (includes Italy, France, U.S., Australia, and Japan)	1966–2017	Older adults	Vaccination reduced the risk of experiencing influenza in a single season from 6.0% to 2.4% (risk ratio (RR) 0.42, 95% confidence interval (CI) 0.27 to 0.66)	[23]
Major health consequences	Systematic review and meta-analysis	Global (includes France, U.S., and Germany)	1980–2021	Older adults	Vaccination was associated with a reduced risk of having a stroke and subsequent hospitalization by 16% (OR 0.84, 95% CI: 0.77 to 0.90)	[24]
Mortality	Cohort study	Italy	2009–2017	Adults aged 65 and over	Vaccination decreased an individual’s risk of all-cause mortality by 13% (HR 0.87, 95% CI 0.80–0.95) during the 2018/2019 winter season.	[25]
Pneumococcal						
Disease prevention	Systematic review and meta-analysis	Global (Includes U.S., Italy, Germany, and Japan)	2016–2022	Older adults	PCV13 vaccine efficacy was reported to be 75% against PCV13-type invasive pneumococcal disease (IPD) and 45% against PCV13-type pneumococcal pneumonia (PP). The pooled PPSV23 vaccine effectiveness was 45% (95% CI: 37%, 51%) against PPSV23-type IPD and 18% (95% CI: −4%, 35%) against PPSV23-type PP.	[26,27]
Major health consequences	Systematic review and meta-analysis	U.S.	Database inception–2022	Older adults (varies by study)	Vaccination was associated with a decline in the incidence of myocardial infarctions (HR, 0.73, 95% CI: 0.56–0.96) compared with no vaccination. Incidence of stroke did not differ between the two groups.	[28]
Mortality	Systematic review and meta-analysis	U.S.	Database inception—2022	Older adults (varies by study)	Vaccination was associated with a decrease in the risk of all-cause mortality among adults (HR: 0.76, 95% CI: 0.66 to 0.87) compared with no vaccination. Incidence of cardiovascular mortality did not differ between the two groups.	[28]
RSV						
Disease prevention	Randomised, placebo-controlled phase III clinical trial	Global (Northern and Southern hemispheres)	2021–2022	Adults aged 60 and older (healthy and with coexisting conditions)	Vaccines had protective efficacy against lower respiratory tract disease of 82.6% (95% CI, 57.9 to 94.1) and against severe lower respiratory disease of 94.1% (95% CI, 62.4 to 99.9). Among participants with coexisting conditions, vaccine efficacy was 94.6% (95% CI, 65.9 to 99.9)	[29]
	Randomised, placebo-controlled phase III clinical trial	Global (Sites in Argentina, Canada, Finland, Japan, the Netherlands, South Africa, and U.S.)	2021–2022	Adults aged 60 and older	Vaccine efficacy against RSV-related lower respiratory illness with at least two signs or symptoms was 65.1%, and vaccine efficacy against RSV-related lower respiratory illness with at least three signs or symptoms was 88.9% in adults aged 60 and older	[30]
Major health consequences	Evidence is not yet available directly linking RSV vaccination to major health consequences and mortality reduction					
Mortality						
Herpes Zoster						
Disease prevention	Systematic review and meta-analysis	Multi-continent	Database inception–2022	Adults 60 years or older (healthy and immuno-compromised)	Recombinant zoster (RZV) vaccine efficacy against HZ was 94% (95% CI: 87–97%) and ZVL vaccine efficacy was 62% (95% CI: 23–82%). In immunocompromised subjects, RZV vaccine efficacy was 60% (95% CI: 49–69%).	[31]
	Long-term follow-up study	Global (Including France, Germany, Italy, Japan, and U.S.)	2016–2021	Adults 50 years or older	Efficacy of RZV against HZ was up to 73.2% ten years post-vaccination.	[32]
Major health consequences	Randomised, placebo-controlled phase III clinical trial	Multi-continent	2010–2015	Adults aged 70 years and older	Vaccine efficacy against herpes zoster was 89.8% (95% CI, 84.2 to 93.7) and was similar in participants 70 to 79 years of age (90.0%) and participants 80 years of age or older (89.1%). Pooled analyses of trial data from participants 70 years of age or older showed that vaccine efficacy against postherpetic neuralgia was 88.8% (95% CI, 68.7 to 97.1)	[33]
	Retrospective case–control study	U.S.	2010–2020	Patients aged 18 years and older who received care at a Veterans Affairs facility	HZ cases (average age 71) who had received any vaccination against HZ had a significantly decreased odds of experiencing stroke in the 30 days following HZ infection: RZV vaccine (OR, 0.57, 95% CI, 0.46–0.72) or ZVL vaccine (OR, 0.77, 95% CI, 0.65–0.91)	[34]
	Cohort study	U.S. (California)	2020	Adults aged 50 years and older	Vaccine recipients aged 50 and older had a 16% lower risk of COVID-19 diagnosis and a 32% lower risk of related hospitalization	[35]
Mortality *	Static multi-cohort Markov model	Germany	2015 (lifetime horizon)	Adults aged 50 years and older	Although HZ does not usually cause death, vaccination is associated with avoided HZ-related mortality, which increases with expanded coverage.	[36]

* HZ does not usually cause death, most studies of vaccine efficacy do not consider this outcome.

#### 3.2.2. Value of Adult Immunisation Programmes for Reducing Disease Transmission

There is a small body of literature exploring the effects of adult immunisation programmes on broader disease transmission dynamics. We identified two studies which reported directly on the additional health benefits accrued to unvaccinated populations due to adult immunisation programmes. A study of the community effects of influenza vaccination in Australia and South Africa found that, compared with no vaccination, vaccination of 15% of the population (prioritising HIV-positive individuals, adults aged 65 and older; and young children) could decrease the annual rate of symptomatic infection by over 47% and deaths by over 55% in both communities [37]. 

### 3.3. Value of Adult Immunisation Programmes for Healthcare Systems

There is good evidence that adult immunisation programmes avert substantial costs to healthcare systems and are cost-effective, and that increased uptake or coverage expansion would be more cost-effective for healthcare systems. This is summarized in Table 4.

**Table 4:** Findings on the value of adult vaccination programmes to healthcare systems.

Vaccination	Study Type	Geography	Timeframe	Population	Findings	Reference
Influenza						
Cost-effectiveness and cost savings	Systematic review	North America (U.S. and Canada)	1980–2016	Adults 19 years and older	56% of age-based adult influenza immunisation programmes resulted in net cost savings, and 100% of age-based adult influenza immunisation programmes reported a cost-per-QALY of less than USD 50,000	[38]
	Retrospective cohort study	Australia	2017–2019	Older adults (median age: 80)	Influenza vaccination in 100% of eligible inpatients led to an overall reduction of AUD 26,736 in hospitalisation costs (mean reduction of AUD 477 per patient). This represents an offset of AUD 18 for the influenza vaccine intervention, and net savings of AUD 459 per patient vaccinated (i.e., AUD 328 per patient per year).	[39]
	Cost analysis within a retrospective cohort study	Germany	2015–2016	Adults 60 years and older	Influenza vaccination appeared as cost-saving, with lower disease-related health care costs of EUR 178.87 (95% CI EUR 240.03; EUR 117.17) per individual. Cost-savings mainly result from averted hospital inpatient and emergency care.	[40]
Increasing uptake	Markov decision analysis model	U.S.	10-year model time horizon	Adults 65 years and older	Over 10 years, interventions to increase vaccine uptake would result in 60,920 fewer influenza cases, at an estimated cost of USD 512 per QALY gained.	[41]
Expansion	Cost-benefit analysis	Australia	2017–2018	Adults aged 50–64 years	Expansion of the national influenza immunization programme to adults aged 50–64 years is estimated to be cost-saving for the government, with an estimated AUD 8.03 million saved and an incremental benefit-cost ratio of 1.40. Cost-savings are mostly due to reduced acute myocardial infarction hospitalizations.	[42]
	Cost-effectiveness analysis	France, Italy, and Poland (and Romania)	9-month model time horizon	General population	Universal vaccination targeting the general population was more cost-effective than the vaccination of priority groups alone, which were also considered to be cost-effective or cost-saving.	[43]
Pneumococcal						
Cost-effectiveness and cost savings	Systematic review	Global (including Brazil, France, Germany, Japan, Italy, and U.S.)	Database inception–2016	Adults 60 years and older	Most studies considered PPV23 cost-effective (less than USD 50,000 per LYG or QALY) and sometimes cost-saving (results ranging from cost-saving to USD 84,636/QALY), driven by reduced hospitalization costs, improved quality of life and increased life expectancy.	[44]
	Systematic review	North America (U.S. and Canada)	1980–2016	Adults 65 and older, high-risk adults aged 19–64 years	31% of age-based adult pneumococcal immunisation programmes were found to result in net cost savings. 78% were cost-effective at a threshold of USD 50,000/QALY, and 100% at USD 100,000/QALY	[38]
Increasing uptake	Markov state transition model	Australia	10-year horizon	Adults 65 years and older with no history of acute coronary syndrome	Increasing vaccination coverage from 50% to 100% was estimated to result in cost-savings of AUD 179 per person, with a QALY gain of 0.0075	[45]
Expansion	Cost-effectiveness analysis	U.S.	Lifetime model time horizon	Adults aged 50–64 with chronic kidney diseases	Expanding current recommendations to include adults aged 50–64 with chronic kidney disease in immunisation programmes would be cost-effective at a threshold of USD 100,000 per QALY, with a cost-effectiveness ratio of USD 38,000/QALY compared with no vaccination.	[46]
RSV						
Cost-effectiveness and cost savings	Systematic review	Global (including Australia and U.S.)	2000–2020	Pregnant women and older adults (65 years and older)	The cost-effectiveness of maternal vaccination was USD 1766–5857 PPP 2018 per disability-adjusted life-years (DALYs) for Global Alliance for Vaccines and Immunisation (Gavi)-eligible countries, and USD 81.5 PPP 2018 for high-income settings. Vaccination in the elderly could potentially be as cost-effective as influenza vaccines, depending on vaccine characteristics and target population (ICER range USD 6886.30 to 189,282.90 PPP 2018 per QALY gained).	[47]
	Cost-effectiveness analysis	U.S.	1 year time horizon	Adults 60 years and older	A vaccine with 50% efficacy and coverage matching that of influenza vaccination is likely to be cost-effective at prices ranging from USD 73.54 to USD 298.79 per vaccination, depending on the epidemiology data used and the willingness-to-pay threshold considered.	[48]
Increasing uptake	N/A					
Expansion	N/A					
Herpes Zoster						
Cost-effectiveness and cost savings	Systematic review	U.S.	1980–2016	Adults 60 years and older	71% of HZ immunisation programmes using ZVL vaccines reported a cost-per-QALY of less than USD 100,000	[38]
	Systematic review	U.S.	2015–2021	Older adults varies by study	100% of studies comparing RZV with no vaccine found RZV to be a cost-effective strategy to prevent shingles and post-herpetic neuralgia. Variation in cost-effectiveness noted between age categories	[49]
	Literature review	Global (including Germany, Japan, and U.S.)	2017–2022	Older adults varies by study	RZV vaccination was cost-effective in 15 out of the 18 included studies in comparison to either no vaccination (or prior vaccination with ZVL)	[50]
Increasing uptake	None identified					
Expansion	Systematic review	U.S.	2015–2021	Older adults	Vaccination was cost-saving in adults aged 60 and over, and cost-effective in adults aged 50 and over with a cost-per-QALY of USD 14,916 per QALY gained	[49]

### 3.4. Value of Adult Immunisation Programmes to Society

#### 3.4.1. Productivity Value of Adult Immunisation Programmes

There is evidence that vaccines avert major productivity losses, and that expansions of vaccination coverage would produce net gains for governments as a result of averted productivity losses among immunised populations (and the resulting fiscal implications, including increased income tax revenue as a result). This is summarized in Table 5.

There is limited evidence of the productivity value to caregivers. Three studies reported results incorporating productivity effects on caregivers. A cost-effectiveness analysis of influenza immunisation programmes in South Africa including adults aged 65 years and older reported productivity losses averted amongst their caregivers, and found the programme to be cost-effective [51]. Similar analyses of pneumococcal and HZ immunisation programmes in adults aged 60 and over (some of whom had underlying conditions) in Japan reported productivity costs averted amongst caregivers and found the programmes cost-effective [52,53]. 

We did not identify any evidence valuing the productivity gains of vaccination amongst older adults in terms of the value of the informal care they themselves contribute (e.g., to grandchildren).

**Table 5:** Findings on the productivity value of adult vaccination programmes in immunised populations.

Study Type	Geography	Time Frame	Population	Findings	Reference
Influenza					
Cost–benefit analysis	Italy	1-year time horizon	Adults aged 30 to 65 years	A vaccination strategy resulting in a reduction of the number of infected people by 200,000 (10% of current levels) would reduce productivity losses by EUR 111 million and increase tax revenue by nearly EUR 18 million annually.	[54]
Observational cohort study	Italy	2017–2018	Healthy, working-age adults	56.4% reduction in average sick-leave days per person compared with unvaccinated individuals and a net cost saving of EUR 314 per person when considering the costs of vaccination and absenteeism.	[55]
Pneumococcal					
Cost–benefit analysis	Italy	1-year time horizon	Adults aged 30 to 65 years	A vaccination strategy resulting in a reduction of the number of infected people by 9000 (10% of current levels) would decrease productivity losses by EUR 124 million and increase tax revenue by EUR 24 million annually. Investment in this strategy would yield average per capita benefits of 16.2 times the value of the investment in terms of productivity impact and 3.1 times the value of the investment in terms of tax impact over the 1-year time horizon.	[54]
RSV					
None identified					
Herpes Zoster					
Cost–benefit analysis	Italy	1-year time horizon	Adults aged 30 to 65 years	A vaccination strategy resulting in a reduction in the number of individuals infected with HZ from 6400 to 6000 and with PHN from 1050 to 750 would result in a total annual reduction in productivity loss of EUR 640,000 and increase in tax revenue of EUR 63,000. Investment in this strategy would yield average per capita benefits of 20.0 times the value of the investment in terms of productivity impact and 1.7 times the value of the investment in terms of tax impact.	[54]

#### 3.4.2. Social Equity Value of Adult Immunisation Programmes 

Vaccine programmes can contribute to improved health equity within countries, as well as a reduction in the financial risk associated with vaccine-preventable diseases, which is inequitably distributed. A systematic review of ‘equity-informative’ economic evaluations (i.e., evaluations which consider equity dimensions) of vaccines concluded that both the introduction of vaccine programmes and expanded vaccine coverage resulted in mortality reductions and financial risk benefits, which were relatively larger in subpopulations with higher disease burdens and lower vaccination coverage—in particular, poorer income groups and those living in rural areas [56]. A modelling study exploring the expected equity effects of ten vaccines, including influenza and pneumococcal vaccines, in forty-one low- and middle-income countries between 2016 and 2030 estimated that the largest effects on averted deaths and cases of medical impoverishment would be in the lowest income quartile of the population across vaccines and countries [57]. 

Expanded vaccine coverage could further increase the equity value of vaccines, as well as their broader health and economic value to society. A modelling study in the US found that expanding the pneumococcal vaccination recommendation to all adults over the age of 50 (compared to the current recommendation of vaccination for adults aged 65 and older and high-risk adults) would reduce inequity in the pneumococcal disease burden between black and non-black populations [58]. This is because, in the US, black populations aged 50 to 65 have a higher prevalence of risk factors, a higher probability of undiagnosed underlying medical conditions, and a greater risk of pneumococcal disease [59]. The expanded recommendation would produce greater overall health and economic benefits, and be more cost-effective [58]. 

#### 3.4.3. Role of Adult Immunisation Programmes in the Fight against Anti-Microbial Resistance (AMR)

Vaccines can affect antimicrobial resistance (AMR) directly via a reduction in the organisms and strains carrying resistant genes specifically targeted by a vaccine, and indirectly through a reduction in illnesses which require treatment with antibiotics. Evidence suggests that pneumococcal vaccines are associated with a direct and significant reduction in the number of antibiotic-resistant invasive pneumococcal disease episodes in vaccinated groups compared with unvaccinated controls [12,60,61,62]. While antiviral vaccines (e.g., influenza and RSV) do not directly affect organisms causing antibiotic-resistant disease, they reduce the incidence of illnesses for which antibiotics are inaccurately prescribed, as well as the risk of secondary bacterial infections which require antibiotic treatment. Significant reductions (11–50%) in the use of antibiotics have been observed in influenza-vaccinated adults compared with controls [61]. One case–control study in Australia which assessed the effects of the influenza vaccine on antibiotic prescription for influenza-like-illness recorded a 22–23% reduction the likelihood of antibiotic prescribing in low-risk adults (aged 40–64 years and without comorbidities) [63]. New vaccines targeting respiratory pathogens, such as RSV, would not only prevent the viral disease, but could potentially curtail subsequent antibiotic use and, consequently, induce AMR [64]. 

## 4. Discussion

This study shows that there is a substantial evidence base on the value of adult immunisation programmes, but the availability of evidence varies by value element and by vaccine. Evidence availability was greatest for the ‘narrow’ benefits of immunisation programmes: the quality of life and mortality benefits to vaccinated individuals and the cost-offsets to healthcare systems. The availability of evidence for ‘broader’ societal value elements (such as transmission value, carer productivity, and impact on social equity and antimicrobial resistance prevention) varied. No evidence was identified relating to the broader value elements of macroeconomic effects, value to other interventions, or effects on the quality of life of caregivers.

Quality of life and mortality were well-evidenced for each of the vaccines of focus, with demonstrated effectiveness in older adults and at-risk populations. While there is some evidence that some vaccines may be less effective in the oldest populations, due to the progressive decline of immunity with age, we found evidence of immune response and efficacy even in the frailest and most immunocompromised populations. This is important given that these populations often suffer the most severe consequences of vaccine-preventable diseases [33,36]. These diseases can act in a costly ‘vicious cycle’, accelerating frailty and in turn making individuals more vulnerable to the health consequences of vaccine-preventable diseases [65,66]. A well-functioning immune system (supported by adherence to vaccination schedules) can thus delay the acceleration of frailty to disability, highlighting the importance of vaccinating the oldest populations on an individual and societal level. Furthermore, new strategies are being developed to improve vaccine efficacy in adults aged 80 and over [67]. With ageing global populations and higher healthcare resource utilisation and costs amongst older patients, as well as higher rates of co-morbidities, vaccines which are effective in—and widely used by—the oldest populations will be critical [22,68].

The value of adult immunisation programmes in off-setting costs to health systems is also well-evidenced. We found that all of the adult immunisation programmes considered are at least cost-effective (or have the potential to be, in the case of RSV) [38,47,48,49] and can result in net cost savings to the healthcare system [39,40,45]. Interventions to increase uptake can also be cost-effective [41] and even increase cost-effectiveness [45], potentially due to economies of scale in delivery, considering the relatively low variable costs compared with fixed costs, and in some cases population-level transmission effects (herd protection) [69,70]. Expanding access to a broader adult population can potentially generate more value, especially given that the burden of vaccine-preventable diseases and rates of chronic diseases amongst younger age groups is projected to rise [71].

Vaccines provide important societal value. The most well-evidenced broader value element was the impact on productivity of the vaccinated, evidenced in 70% of our countries and for all of the vaccines of focus. Adult immunisation programmes provide a positive return on investment in the form of increased productivity (and subsequently, increased income tax revenues), which can outweigh their costs many times over [54]. It is important to note, however, that the included studies only considered the productivity impacts of absenteeism, but not the potential effects of some vaccines, like pneumococcal and herpes zoster vaccines, on presenteeism [72]. Additionally, they do not consider productivity effects on informal care delivered, for example, by older adults [73], nor the potential productivity value for younger adults in high-risk groups.

In recent years, academic publications have increasingly evidenced diverse elements of socio-economic value associated with adult vaccination. For example, in comparison with the studies on pneumococcal vaccination published between 2010 and 2016 reviewed by Cafiero Fonseca et al. [12], which identified evidence pertaining to six of the value elements in the framework, our research additionally identified evidence relating to two more value elements: social equity value and AMR prevention value. Whilst our results show that there is growing recognition of the broad societal value of vaccination, it is also clear that evidence gaps remain. Our review identified a paucity of evidence relating to broader value elements like AMR prevention and carer productivity, and we found no evidence relating to macroeconomic effects, value to other interventions, or effects on the quality of life of caregivers in any country in our sample. This can be explained partly by the methodological challenges involved in collecting and analysing evidence of broader value, and partly by the ‘narrow’ decision-making frameworks used to evaluate immunisation programmes [12,13,14,16]. 

It is important to recognise the broader value of vaccines, as oversight of some value elements risks the undervaluation of vaccination programs. For example, vaccination plays an important role in preventing AMR; thus, the exclusion of AMR prevention value in cost-effectiveness analyses may yield less favourable cost-effectiveness results [5,62,63]. Investment in the development of novel antimicrobial vaccines can help to protect against the future progression of AMR, a value which may be complex to quantify given the technical challenges of measuring direct and indirect effects of vaccines on AMR and the lack of data availability [5,61,74]. More comprehensive approaches to the design of cost-effectiveness studies, which simultaneously evaluate several criteria and in-corporate qualitative judgements where quantitative data may not be available, are increasingly possible and may be necessary to capture the broader value of vaccines [75].

The understanding and quantification of these broader value elements, and their incorporation into cost-effectiveness analyses, have important policy implications. For example, given that socioeconomically disadvantaged sub-populations have higher rates of underlying medical conditions—and subsequently a higher risk of vaccine-preventable disease and complications—the benefits of expanding adult immunisation programmes are particularly concentrated in these sub-populations [56,57,58,59]. Capturing these context-dependent social equity considerations in cost-effectiveness analysis can enable the design of vaccine policies with the potential to reduce inequity in the distribution- and economic and societal burden- of vaccine-preventable diseases.

Existing methods and tools need to be adjusted to integrate these additional (broader) benefits for which empirical evidence has become increasingly available in recent years [17]. More experimental and observational studies are also needed to expand the evidence base for the broader benefits of vaccination, beyond those included in existing conceptual frameworks. Gessner et al. [76] call for an inventory of evidence and regular monitoring of progress on completeness. Our research is a step in that direction. 

The strengths of this review include a rigorous and structured search and literature evaluation by more than one person, enabling a comprehensive mapping of availability of evidence by value element in our selected countries. To our knowledge, this is the first use of the Broader Value of Vaccines framework by Bell et al. [16] to evaluate evidence availability and identify gaps by value element across the four selected immunisation programmes. We included a diverse set of countries and programmes to enhance the generalisability of the results and enable a better understanding of some contextual differences; however, our study has some limitations. Given the breadth of our scope, there is a trade-off with the systematic and comprehensive inclusion of evidence. We did not conduct a meta-analysis given the diversity of outcomes covered by the value elements considered and due to methodological heterogeneity in the studies included. Risk of bias was mitigated by excluding evidence from the lowest tiers of the evidence hierarchy. While this study includes a set of ten countries with various vaccination schemes, the generalisability of specific quantitative results is limited, as the magnitude of effects considered depends on contextual factors such as the uptake of both adult and child immunisation programmes [12]. Further, our search was limited to the English literature. Therefore, public health reports or national studies that have not been published as scientific papers in English, but which may contain information on vaccination and quantification of its economic/societal value, are not reflected in our results. We also note that the majority of reported evidence pertains to the benefits of adult immunisation programmes, which may suggest publication bias. Finally, the evidence on RSV is limited given its novelty at the time of writing. 

The combination of countries and immunisation programmes selected aims to provide a comprehensive picture of the recognition of the value of adult immunisation across a global context, balancing the breadth of adult vaccination programmes (e.g., those targeting fatal vs. non-fatal illnesses, which are less of a priority in low-income countries) with countries geographical (relevant for incidence rates of certain vaccine-preventable diseases), income, and data availability contexts. Our country selection includes ten diverse countries: while some middle-income countries were included, the implementation of immunisation programmes—and consequently, data availability—is often limited in low-income countries [17]. Further research and investment are needed to specifically evaluate adult immunisation programmes in low-income countries and to generate focused analyses, for example, using the framework proposed by Hutubessy et al. [17], to help to improve the sustainability and equity of adult immunisation programmes. 

## 5. Conclusions

Robust evidence exists demonstrating that adult immunisation programs can counter the increasing burden of vaccine-preventable diseases while being cost-effective for health systems. Expanding access to a broader adult population can generate greater value for society and net cost savings for healthcare systems. Further research evidencing the broader elements of the value of immunisation programmes where we identified gaps is essential to inform better decision-making and targeted policy interventions that aim to optimise the coverage of adult immunisation programmes and maximise their value to individuals, healthcare systems, and society. 

## Figures and Tables

**Table 1:** Value domains and value elements according to the Broader Value of Vaccines framework.

Value Domain	Value Element *	Broad or Narrow?	Definition
Population health	Impact on quality of life of vaccinated	Narrow	Value of effects on the physical, mental, emotional, and social functioning of vaccinated individuals
	Impact on mortality of vaccinated	Narrow	Value of effects on life expectancy or life-years saved of vaccinated individuals
	Impact on quality of life of carers	Broad	Value of effects on the physical, mental, emotional, and social functioning of caregivers of vaccinated individuals
	Transmission value	Broad	Value of effects on disease transmission patterns and associated quality of life and mortality effects in non-vaccinated individuals
Healthcare systems	Cost offsets to healthcare system	Narrow	Value of effects on net resource use by healthcare systems in providing care to vaccinated individuals, i.e., the value of resources spent on avoidable illness (opportunity cost)
	Value for other interventions	Broad	Value of increasing the cost-effectiveness of other non-vaccine interventions
Society	Impact on productivity of vaccinated	Broad	Value of effects on net time spent at work/in informal care and the level of productivity of vaccinated individuals, and associated fiscal impact
	Impact on carer productivity	Broad	Value of effects on net time spent at work and the level of productivity at work of caregivers of vaccinated individuals
	Social equity value	Broad	Value of effects on disparities in the distribution of health across the population
	AMR ** prevention value	Broad	Value of slowing the rate of development and transmission of resistant bacterial, fungal, parasitic, and viral infections, and associated effects on quality of life and mortality
	Macroeconomic effects	Broad	Value of effects on the macroeconomy beyond productivity, e.g., effects on the value of trade during major outbreaks. ***

* In research assessing consideration of broader value by HTA agencies, the Broader Value of Vaccines framework also includes the element ‘Burden of disease value’, intended to reflect the prioritisation by some of these bodies of interventions impacting diseases with high disease burdens [16,20]. This element is not relevant for the purpose of this paper of assessing the consideration of the value of vaccines in research studies. ** AMR = Antimicrobial resistance. *** Macroeconomic effects not predicted to be relevant for vaccines of focus given low likelihood of major outbreaks.

**Table 2:** Percentage of sample for which country-specific evidence of positive impact on value element was identified.

Value Domain	Population Health				Healthcare System		Society				
Value Element	Impact on Quality of Life of Vaccinated	Impact on Mortality of Vaccinated	Impact on Quality of Life of Carers	Transmission Value	Cost Offsets to Healthcare System	Value to Other Interventions	Impact on Productivity of Vaccinated	Impact on Carer Productivity	Social Equity Value	AMR * Prevention Value	Macroeconomic Effects
	Narrow	Narrow	Broad	Broad	Narrow	Broad	Broad	Broad	Broad	Broad	Broad
Influenza	100	100	0	50	80	0	60	20	0	10	0
Pneumococcal	60	50	0	30	60	0	40	10	10	0	0
RSV	60	10	0	10	10	0	0	0	0	0	0
HZ	70	30	0	NA	40	0	30	10	0	0	0

* AMR = Antimicrobial resistance.

## Data Availability

Data are contained within the article or Supplementary Materials.

## Supplementary Materials

The following supporting information can be downloaded at: https://www.mdpi.com/article/10.3390/vaccines12080852/s1, Table S1. Adult vaccination recommendations in selected countries; Table S2: Full list of papers reviewed and included in evidence mapping for country matrices.

## Appendix A

The following search strategy was used in the PubMed database to identify relevant research: ((influenza[Title/Abstract]) OR (pneumo*[Title/Abstract]) OR (zoster[Title/Abstract]) OR (RSV[Title/Abstract]) OR (respiratory syncytial virus[Title/Abstract])) AND ((vaccin*[Title/Abstract]) OR (burden[Title/Abstract]) OR (impact[Title/Abstract])) AND ((australia) OR (brazil) OR (france) OR (germany) OR (Italy) OR (japan) OR (poland) OR (south africa) OR (thailand) OR (united states)). The study design is summarised in Table A1.

**Table A1:** PICO Table.

Population	Adults ≥ 18 years
Intervention	Influenza, pneumococcal, HZ, and RSV vaccines; evidence on the burden caused by the diseases these vaccines target also included.
Comparator	For vaccine intervention, no vaccine.
Outcomes	Any: e.g., health outcomes, societal economic outcomes

## Appendix B

*Country Matrices of Evidence Availability for Value of Adult Immunisation Programmes*

**Table A2:** Australia *.

Value Domain	Population Health				Healthcare System		Society				
Value Element	Impact on Quality of Life of Vaccinated	Impact on Mortality of Vaccinated	Impact on Quality of Life of Carers	Transmission Value	Cost Offsets to Healthcare System	Value to Other Interventions	Impact on Productivity of Vaccinated	Impact on Carer Productivity	Social Equity Value	AMR Prevention Value	Macroeconomic Effects
Influenza	X	X		X	X		X			X	
Pneumococcal	X	X		X	X						
RSV	X										
HZ	X										
All vaccines	X	X		X	X		X			X	

* X = evidence available for value domain.

**Table A3:** Brazil *.

Value Domain	Population Health				Healthcare System		Society				
Value Element	Impact on Quality of Life of Vaccinated	Impact on Mortality of Vaccinated	Impact on Quality of Life of Carers	Transmission Value	Cost Offsets to Healthcare System	Value to Other Interventions	Impact on Productivity of Vaccinated	Impact on Carer Productivity	Social Equity Value	AMR Prevention Value	Macroeconomic Effects
Influenza	X	X			X		X				
Pneumococcal											
RSV	X										
HZ	X										
All vaccines	X	X			X		X				

* X = evidence available for value domain.

**Table A4:** France *.

Value Domain	Population Health				Healthcare System		Society				
Value Element	Impact on Quality of Life of Vaccinated	Impact on Mortality of Vaccinated	Impact on Quality of Life of Carers	Transmission Value	Cost Offsets to Healthcare System	Value to Other Interventions	Impact on Productivity of Vaccinated	Impact on Carer Productivity	Social Equity Value	AMR Prevention Value	Macroeconomic Effects
Influenza	X	X		X	X						
Pneumococcal	X			X							
RSV											
HZ	X										
All vaccines	X	X		X	X						

* X = evidence available for value domain.

**Table A5:** Germany *.

Value Domain	Population Health				Healthcare System		Society				
Value Element	Impact on Quality of Life of Vaccinated	Impact on Mortality of Vaccinated	Impact on Quality of Life of Carers	Transmission Value	Cost Offsets to Healthcare System	Value to Other Interventions	Impact on Productivity of Vaccinated	Impact on Carer Productivity	Social Equity Value	Amr Prevention Value	Macroeconomic Effects
Influenza	X	X			X		X				
Pneumococcal	X	X		X	X		X				
RSV	X										
HZ	X	X			X						
All vaccines	X	X		X	X		X				

* X = evidence available for value domain.

**Table A6:** Italy *.

Value Domain	Population Health				Healthcare System		Society				
Value Element	Impact on Quality of Life of Vaccinated	Impact on Mortality of Vaccinated	Impact on Quality of Life of Carers	Transmission Value	Cost Offsets to Healthcare System	Value to Other Interventions	Impact on Productivity of Vaccinated	Impact on Carer Productivity	Social Equity Value	AMR Prevention Value	Macroeconomic Effects
Influenza	X	X			X		X				
Pneumococcal					X		X				
RSV	X										
HZ	X				X		X				
All vaccines	X	X			X		X				

* X = evidence available for value domain.

**Table A7:** Japan *.

Value Domain	Population Health				Healthcare System		Society				
Value Element	Impact on Quality of Life of Vaccinated	Impact on Mortality of Vaccinated	Impact on Quality of Life of Carers	Transmission Value	Cost Offsets to Healthcare System	Value to Other Interventions	Impact on Productivity of Vaccinated	Impact on Carer Productivity	Social Equity Value	AMR Prevention Value	Macroeconomic Effects
Influenza	X	X		X	X		X				
Pneumococcal	X	X			X		X	X			
RSV	X										
HZ	X	X			X		X	X			
All vaccines	X	X		X	X		X	X			

* X = evidence available for value domain.

**Table A8:** Poland *.

Value Domain	Population Health				Healthcare System		Society				
Value Element	Impact on Quality of Life of Vaccinated	Impact on Mortality of Vaccinated	Impact on Quality of Life of Carers	Transmission Value	Cost Offsets to Healthcare System	Value to Other Interventions	Impact on Productivity of Vaccinated	Impact on Carer Productivity	Social Equity Value	AMR Prevention Value	Macroeconomic Effects
Influenza	X	X									
Pneumococcal											
RSV											
HZ											
All vaccines	X	X									

* X = evidence available for value domain.

**Table A9:** South Africa *.

Value Domain	Population Health				Healthcare System		Society				
Value Element	Impact on Quality of Life of Vaccinated	Impact on Mortality of Vaccinated	Impact on Quality of Life of Carers	Transmission Value	Cost Offsets to Healthcare System	Value to Other Interventions	Impact on Productivity of Vaccinated	Impact on Carer Productivity	Social Equity Value	AMR Prevention Value	Macroeconomic Effects
Influenza	X	X		X	X		X	X			
Pneumococcal	X	X			X		X				
RSV	X										
HZ											
All vaccines	X	X		X	X		X	X			

* X = evidence available for value domain.

**Table A10:** Thailand *.

Value Domain	Population Health				Healthcare System		Society				
Value Element	Impact on Quality of Life of Vaccinated	Impact on Mortality of Vaccinated	Impact on Quality of Life of Carers	Transmission Value	Cost Offsets to Healthcare System	Value to Other Interventions	Impact on Productivity of Vaccinated	Impact on Carer Productivity	Social Equity Value	AMR Prevention Value	Macroeconomic Effects
Influenza	X	X			X						
Pneumococcal											
RSV											
HZ											
All vaccines	X	X			X						

* X = evidence available for value domain.

**Table A11:** United States *.

Value Domain	Population Health				Healthcare System		Society				
Value Element	Impact on Quality of Life of Vaccinated	Impact on Mortality of Vaccinated	Impact on Quality of Life of Carers	Transmission Value	Cost Offsets to Healthcare System	Value to Other Interventions	Impact on Productivity of Vaccinated	Impact on Carer Productivity	Social Equity Value	AMR Prevention Value	Macroeconomic Effects
Influenza	X	X		X	X		X	X			
Pneumococcal	X	X			X				X		
RSV	X	X		X	X						
HZ	X	X			X		X				
All vaccines	X	X		X	X		X	X	X		

* X = evidence available for value domain.

## References

[1] Rémy V., Zöllner Y., Heckmann U. (2015). Vaccination: The Cornerstone of an Efficient Healthcare System. J. Mark. Access Health Policy.

[2] Disease Elimination and Eradication. https://historyofvaccines.org/vaccines-101/what-do-vaccines-do/disease-elimination-and-eradication.

[3] Carter A., Msemburi W., Sim S.Y., Gaythorpe K.A.M., Lambach P., Lindstrand A., Hutubessy R. (2024). Modeling the Impact of Vaccination for the Immunization Agenda 2030: Deaths Averted Due to Vaccination against 14 Pathogens in 194 Countries from 2021 to 2030. Vaccine.

[4] UNICEF SDG Goal 3: Good Health and Well-Being. https://data.unicef.org/sdgs/goal-3-good-health-wellbeing/.

[5] Buchy P., Ascioglu S., Buisson Y., Datta S., Nissen M., Tambyah P.A., Vong S. (2020). Impact of Vaccines on Antimicrobial Resistance. Int. J. Infect. Dis..

[6] Greenwood B. (2014). The Contribution of Vaccination to Global Health: Past, Present and Future. Philos. Trans. R. Soc. B Biol. Sci..

[7] Vanderslott S., Roser M. Vaccination. https://ourworldindata.org/vaccine-preventable-diseases.

[8] Institute for Health Metrics and Evaluation (2018). Global Burden of Disease Study 2017 (GBD 2017) Cause-Specific Mortality 1980–2017.

[9] World Health Organisation (2023). WHO Recommendations for Routine Immunization—Summary Tables March 2023.

[10] World Health Organisation (2023). Vaccination Schedule for Pneumococcal Disease 2023.

[11] World Health Organisation (2024). Influenza Vaccination Coverage 2024.

[12] Cafiero-Fonseca E.T., Stawasz A., Johnson S.T., Sato R., Bloom D.E. (2017). The Full Benefits of Adult Pneumococcal Vaccination: A Systematic Review. PLoS ONE.

[13] Beck E., Biundo E., Devlin N., Doherty T.M., Garcia-Ruiz A.J., Postma M., Sheikh S., Smela B., Toumi M., Wasem J. (2022). Capturing the Value of Vaccination within Health Technology Assessment and Health Economics: Literature Review and Novel Conceptual Framework. Vaccine.

[14] Postma M., Biundo E., Chicoye A., Devlin N., Mark Doherty T., Garcia-Ruiz A.J., Jaros P., Sheikh S., Toumi M., Wasem J. (2022). Capturing the Value of Vaccination within Health Technology Assessment and Health Economics: Country Analysis and Priority Value Concepts. Vaccine.

[15] Mauskopf J., Standaert B., Connolly M.P., Culyer A.J., Garrison L.P., Hutubessy R., Jit M., Pitman R., Revill P., Severens J.L. (2018). Economic Analysis of Vaccination Programs: An ISPOR Good Practices for Outcomes Research Task Force Report. Value Health.

[16] Bell E., Neri M., Steuten L. (2022). Towards a Broader Assessment of Value in Vaccines: The BRAVE Way Forward. Appl. Health Econ. Health Policy.

[17] Hutubessy R., Lauer J.A., Giersing B., Sim S.Y., Jit M., Kaslow D., Botwright S. (2023). The Full Value of Vaccine Assessments (FVVA): A Framework for Assessing and Communicating the Value of Vaccines for Investment and Introduction Decision-Making. BMC Med..

[18] Bärnighausen T., Bloom D.E., Canning D., Friedman A., Levine O.S., O’Brien J., Privor-Dumm L., Walker D. (2011). Rethinking the Benefits and Costs of Childhood Vaccination: The Example of the Haemophilus Influenzae Type b Vaccine. Vaccine.

[19] World Health Organisation (2020). Immunization Agenda 2030: A Global Strategy to Leave No One Behind 2020.

[20] Brassel S., Neri M., O’Neill P., Steuten L. (2021). Realising the Broader Value of Vaccines in the UK. Vaccine X.

[21] Burns P.B., Rohrich R.J., Chung K.C. (2011). The Levels of Evidence and Their Role in Evidence-Based Medicine. Plast. Reconstr. Surg..

[22] Zhang S., Wahi-Singh P., Wahi-Singh B., Chisholm A., Keeling P., Nair H. (2022). RESCEU Investigators Costs of Management of Acute Respiratory Infections in Older Adults: A Systematic Review and Meta-Analysis. J. Glob. Health.

[23] Demicheli V., Jefferson T., Di Pietrantonj C., Ferroni E., Thorning S., Thomas R.E., Rivetti A. (2018). Vaccines for Preventing Influenza in the Elderly. Cochrane Database Syst. Rev..

[24] Tavabe N.R., Kheiri S., Dehghani M., Mohammadian-Hafshejani A. (2023). A Systematic Review and Meta-Analysis of the Relationship between Receiving the Flu Vaccine with Acute Cerebrovascular Accident and Its Hospitalization in the Elderly. BioMed Res. Int..

[25] Lapi F., Marconi E., Gualano M.R., Vetrano D.L., Grattagliano I., Rossi A., Cricelli C. (2022). A Cohort Study on Influenza Vaccine and All-Cause Mortality in Older Adults: Methodological Concerns and Public Health Implications. Drugs Aging.

[26] Farrar J.L., Childs L., Ouattara M., Akhter F., Britton A., Pilishvili T., Kobayashi M. (2023). Systematic Review and Meta-Analysis of the Efficacy and Effectiveness of Pneumococcal Vaccines in Adults 2023. Pathogens.

[27] Berild J.D., Winje B.A., Vestrheim D.F., Slotved H.-C., Valentiner-Branth P., Roth A., Storsäter J. (2020). A Systematic Review of Studies Published between 2016 and 2019 on the Effectiveness and Efficacy of Pneumococcal Vaccination on Pneumonia and Invasive Pneumococcal Disease in an Elderly Population. Pathogens.

[28] Jaiswal V., Ang S.P., Lnu K., Ishak A., Pokhrel N.B., Chia J.E., Hajra A., Biswas M., Matetic A., Dhatt R. (2022). Effect of Pneumococcal Vaccine on Mortality and Cardiovascular Outcomes: A Systematic Review and Meta-Analysis. J. Clin. Med..

[29] Papi A., Ison M.G., Langley J.M., Lee D.-G., Leroux-Roels I., Martinon-Torres F., Schwarz T.F., van Zyl-Smit R.N., Campora L., Dezutter N. (2023). Respiratory Syncytial Virus Prefusion F Protein Vaccine in Older Adults. N. Engl. J. Med..

[30] Walsh E.E., Pérez Marc G., Zareba A.M., Falsey A.R., Jiang Q., Patton M., Polack F.P., Llapur C., Doreski P.A., Ilangovan K. (2023). Efficacy and Safety of a Bivalent RSV Prefusion F Vaccine in Older Adults. N. Engl. J. Med..

[31] Xia Y., Zhang X., Zhang L., Fu C. (2022). Efficacy, Effectiveness, and Safety of Herpes Zoster Vaccine in the Immunocompetent and Immunocompromised Subjects: A Systematic Review and Network Meta-Analysis. Front. Immunol..

[32] Strezova A., Diez-Domingo J., Al Shawafi K., Tinoco J.C., Shi M., Pirrotta P., Mwakingwe-Omari A., Adams M., Ahonen A., Zoster-049 Study Group (2022). Long-Term Protection Against Herpes Zoster by the Adjuvanted Recombinant Zoster Vaccine: Interim Efficacy, Immunogenicity, and Safety Results up to 10 Years After Initial Vaccination. Open Forum. Infect. Dis..

[33] Cunningham A.L., Lal H., Kovac M., Chlibek R., Hwang S.-J., Díez-Domingo J., Godeaux O., Levin M.J., McElhaney J.E., Puig-Barberà J. (2016). Efficacy of the Herpes Zoster Subunit Vaccine in Adults 70 Years of Age or Older. N. Engl. J. Med..

[34] Parameswaran G.I., Wattengel B.A., Chua H.C., Swiderek J., Fuchs T., Carter M.T., Goode L., Doyle K., Mergenhagen K.A. (2023). Increased Stroke Risk Following Herpes Zoster Infection and Protection With Zoster Vaccine. Clin. Infect. Dis..

[35] Bruxvoort K.J., Ackerson B., Sy L.S., Bhavsar A., Tseng H.F., Florea A., Luo Y., Tian Y., Solano Z., Widenmaier R. (2022). Recombinant Adjuvanted Zoster Vaccine and Reduced Risk of Coronavirus Disease 2019 Diagnosis and Hospitalization in Older Adults. J. Infect. Dis..

[36] Curran D., Van Oorschot D., Varghese L., Oostvogels L., Mrkvan T., Colindres R., von Krempelhuber A., Anastassopoulou A. (2017). Assessment of the Potential Public Health Impact of Herpes Zoster Vaccination in Germany. Hum. Vaccines Immunother..

[37] de Boer P.T., Kelso J.K., Halder N., Nguyen T.-P.-L., Moyes J., Cohen C., Barr I.G., Postma M.J., Milne G.J. (2018). The Cost-Effectiveness of Trivalent and Quadrivalent Influenza Vaccination in Communities in South Africa, Vietnam and Australia. Vaccine.

[38] Leidner A.J., Murthy N., Chesson H.W., Biggerstaff M., Stoecker C., Harris A.M., Acosta A., Dooling K., Bridges C.B. (2019). Cost-Effectiveness of Adult Vaccinations: A Systematic Review. Vaccine.

[39] Darmaputra D.C., Zaman F.Y., Khu Y.L., Nagalingam V., Liew D., Aung A.K. (2021). Cost-Analysis of Opportunistic Influenza Vaccination in General Medical Inpatients. Intern. Med. J..

[40] Storch J., Fleischmann-Struzek C., Rose N., Lehmann T., Mikolajetz A., Maddela S., Pletz M.W., Forstner C., Wichmann O., Neufeind J. (2022). The Effect of Influenza and Pneumococcal Vaccination in the Elderly on Health Service Utilisation and Costs: A Claims Data-Based Cohort Study. Eur. J. Health Econ..

[41] Smith K.J., Zimmerman R.K., Nowalk M.P., Lin C.J. (2017). Cost Effectiveness of the 4 Pillars^TM^ Practice Transformation Program to Improve Vaccination of Adults Aged ≥65 Years. J. Am. Geriatr. Soc..

[42] Raj S.M., Chughtai A.A., Sharma A., Tan T.C., MacIntyre C.R. (2019). Cost-Benefit Analysis of a National Influenza Vaccination Program in Preventing Hospitalisation Costs in Australian Adults Aged 50–64 Years Old. Vaccine.

[43] Beresniak A., Rizzo C., Oxford J., Goryński P., Pistol A., Fabiani M., Napoli C., Barral M., Niddam L., Bounekkar A. (2019). Cost-Effectiveness of Public Health Interventions against Human Influenza Pandemics in France: A Methodological Contribution from the FLURESP European Commission Project. Eur. J. Public Health.

[44] Nishikawa A.M., Sartori A.M.C., Mainardi G.M., Freitas A.C., Itria A., Novaes H.M.D., de Soárez P.C. (2018). Systematic Review of Economic Evaluations of the 23-Valent Pneumococcal Polysaccharide Vaccine (PPV23) in Individuals 60 years of Age or Older. Vaccine.

[45] Ren S., Attia J., Li S.C., Newby D. (2021). Pneumococcal Polysaccharide Vaccine Is a Cost Saving Strategy for Prevention of Acute Coronary Syndrome. Vaccine.

[46] Ishigami J., Padula W.V., Grams M.E., Chang A.R., Jaar B., Gansevoort R.T., Bridges J.F.P., Kovesdy C.P., Uchida S., Coresh J. (2019). Cost-Effectiveness of Pneumococcal Vaccination Among Patients With CKD in the United States. Am. J. Kidney Dis..

[47] Treskova M., Pozo-Martin F., Scholz S., Schönfeld V., Wichmann O., Harder T. (2021). Assessment of the Effects of Active Immunisation against Respiratory Syncytial Virus (RSV) Using Decision-Analytic Models: A Systematic Review with a Focus on Vaccination Strategies, Modelling Methods and Input Data. Pharmacoeconomics.

[48] Herring W.L., Zhang Y., Shinde V., Stoddard J., Talbird S.E., Rosen B. (2022). Clinical and Economic Outcomes Associated with Respiratory Syncytial Virus Vaccination in Older Adults in the United States. Vaccine.

[49] Meredith N.R., Armstrong E.P. (2022). Cost-Effectiveness of Herpes Zoster Vaccines in the U.S.: A Systematic Review. Prev. Med. Rep..

[50] Giannelos N., Ng C., Curran D. (2023). Cost-Effectiveness of the Recombinant Zoster Vaccine (RZV) against Herpes Zoster: An Updated Critical Review. Hum. Vaccines Immunother..

[51] Edoka I., Kohli-Lynch C., Fraser H., Hofman K., Tempia S., McMorrow M., Ramkrishna W., Lambach P., Hutubessy R., Cohen C. (2021). A Cost-Effectiveness Analysis of South Africa’s Seasonal Influenza Vaccination Programme. Vaccine.

[52] Igarashi A., Hirose E., Kobayashi Y., Yonemoto N., Lee B. (2021). Cost-Effectiveness Analysis for PCV13 in Adults 60 Years and over with Underlying Medical Conditions Which Put Them at an Elevated Risk of Pneumococcal Disease in Japan. Expert Rev. Vaccines.

[53] Teng L., Mizukami A., Ng C., Giannelos N., Curran D., Sato T., Lee C., Matsuki T. (2022). Cost-Effectiveness Analysis Update of the Adjuvanted Recombinant Zoster Vaccine in Japanese Older Adults. Dermatol. Ther..

[54] Ruggeri M., Di Brino E., Cicchetti A. (2020). Estimating the Fiscal Impact of Three Vaccination Strategies in Italy. Int. J. Technol. Assess. Health Care.

[55] Ferro A., Bordin P., Benacchio L. (2020). Influenza Vaccination and Absenteeism among Healthy Working Adults: A Cost-Benefit Analysis. Ann. Ig. Med. Prev. E Comunità.

[56] Patikorn C., Cho J.-Y., Lambach P., Hutubessy R., Chaiyakunapruk N. (2023). Equity-Informative Economic Evaluations of Vaccines: A Systematic Literature Review. Vaccines.

[57] Chang A.Y., Riumallo-Herl C., Perales N.A., Clark S., Clark A., Constenla D., Garske T., Jackson M.L., Jean K., Jit M. (2018). The Equity Impact Vaccines May Have on Averting Deaths and Medical Impoverishment in Developing Countries. Health Aff..

[58] Wateska A.R., Patricia Nowalk M., Lin C.J., Harrison L.H., Schaffner W., Zimmerman R.K., Smith K.J. (2022). Cost-Effectiveness of Revised US Pneumococcal Vaccination Recommendations in Underserved Minority Adults < 65-Years-Old. Vaccine.

[59] Wateska A.R., Nowalk M.P., Lin C.J., Harrison L.H., Schaffner W., Zimmerman R.K., Smith K.J. (2019). Cost-Effectiveness of Adult Pneumococcal Vaccination Policies in Underserved Minorities Aged 50–64 Years Compared to the US General Population. Vaccine.

[60] Buckley B.S., Henschke N., Bergman H., Skidmore B., Klemm E.J., Villanueva G., Garritty C., Paul M. (2019). Impact of Vaccination on Antibiotic Usage: A Systematic Review and Meta-Analysis. Clin. Microbiol. Infect..

[61] Klugman K.P., Black S. (2018). Impact of Existing Vaccines in Reducing Antibiotic Resistance: Primary and Secondary Effects. Proc. Natl. Acad. Sci. USA.

[62] Wang L.M., Hashiguchi T.C.O., Cecchini M. (2021). Impact of Vaccination on Carriage of and Infection by Antibiotic-Resistant Bacteria: A Systematic Review and Meta-Analysis. Clin. Exp. Vaccine Res..

[63] He W.-Q., Gianacas C., Muscatello D.J., Newall A.T., McIntyre P., Cheng A.C., Liu B. (2022). Effectiveness of Influenza Vaccination in Reducing Influenza-like Illness and Related Antibiotic Prescriptions in Adults from a Primary Care-Based Case-Control Study. J. Infect..

[64] Jansen K.U., Knirsch C., Anderson A.S. (2018). The Role of Vaccines in Preventing Bacterial Antimicrobial Resistance. Nat. Med..

[65] Veronese N., Vassallo G., Armata M., Cilona L., Casalicchio S., Masnata R., Costantino C., Vitale F., Giammanco G.M., Maggi S. (2022). Multidimensional Frailty and Vaccinations in Older People: A Cross-Sectional Study. Vaccines.

[66] Vetrano D.L., Triolo F., Maggi S., Malley R., Jackson T.A., Poscia A., Bernabei R., Ferrucci L., Fratiglioni L. (2021). Fostering Healthy Aging: The Interdependency of Infections, Immunity and Frailty. Ageing Res. Rev..

[67] Bell M., Kutzler M. (2022). An Old Problem with New Solutions: Strategies to Improve Vaccine Efficacy in the Elderly. Adv. Drug Deliv. Rev..

[68] Federici C., Cavazza M., Costa F., Jommi C. (2018). Health Care Costs of Influenza-Related Episodes in High Income Countries: A Systematic Review. PLoS ONE.

[69] World Health Organization (2019). WHO Guide for Standardization of Economic Evaluations of Immunization Programmes.

[70] Sandmann F.G., van Leeuwen E., Bernard-Stoecklin S., Casado I., Castilla J., Domegan L., Gherasim A., Hooiveld M., Kislaya I., Larrauri A. (2022). Health and Economic Impact of Seasonal Influenza Mass Vaccination Strategies in European Settings: A Mathematical Modelling and Cost-Effectiveness Analysis. Vaccine.

[71] CDC (2020). Prevalence of Selected Chronic Conditions Among Children, Adolescents, and Young Adults in Acute Care Settings in Hawai‘i 2020. Prev. Chronic Dis..

[72] Singhal P.K., Makin C., Pellissier J., Sy L., White R., Saddier P. (2011). Work and Productivity Loss Related to Herpes Zoster. J. Med. Econ..

[73] Tur-Sinai A., Teti A., Rommel A., Hlebec V., Lamura G. (2020). How Many Older Informal Caregivers Are There in Europe? Comparison of Estimates of Their Prevalence from Three European Surveys. Int. J. Environ. Res. Public Health.

[74] Yemeke T., Chen H.-H., Ozawa S. (2023). Economic and Cost-Effectiveness Aspects of Vaccines in Combating Antibiotic Resistance. Hum. Vaccines Immunother..

[75] Holm M., Zellweger R.M., Poudyal N., Smith K.H., Joh H.S., Marks F. (2022). Measuring the Link Between Vaccines and Antimicrobial Resistance in Low Resource Settings—Limitations and Opportunities in Direct and Indirect Assessments and Implications for Impact Studies. Front. Trop. Dis..

[76] Gessner B.D., Kaslow D., Louis J., Neuzil K., O’Brien K.L., Picot V., Pang T., Parashar U.D., Saadatian-Elahi M., Nelson C.B. (2017). Estimating the Full Public Health Value of Vaccination. Vaccine.

