# Supplementary material for: A Structured Narrative Literature Review of the Broader Value of Adult Immunisation Programmes
Source: Vaccines (Basel). 2024 Jul 29;12(8):852. doi: 10.3390/vaccines12080852 (PMC11359156; doi:10.3390/vaccines12080852)
Supplement: Supplementary file 1 [file vaccines-12-00852-s001.zip › vaccines-3062173-supplementary.pdf]

vaccines

MDPI

Supplementary Materials

Table S1. Adult vaccination recommendations in selected countries

Country	Vaccine			
	Influenza	Pneumococcal	Herpes Zoster	RSV
Australia	65+ and at risk; >18 in some regions	70+ and 50+ for indigenous communities	70-79	Not included
Brazil	60+ and at risk	Not included	Not included	Not included
France	65+ and at risk	At risk	65-75	Not included
Germany	60+	60+	60+ and at risk >50	60+
Italy	65+ and at risk	65+	65+ and at risk >50	Not included
Japan	65+ and at risk	65+	50+	Not included
Poland	55+	50+*	Not included	Not included
South Africa	65+ and at risk	Not included	Not included	Not included
Thailand	65+ and at risk	Not included	Not included	Not included
United States	18+	65+	50+ and at risk 19+	60+

* Recommended for older adults but not funded by the healthcare system

Source: Data taken from https://vaccine-schedule.ecdc.europa.eu/ and https://immunizationdata.who.int/pages/schedule-by-country/; and specifically (CDC, 2024; EMA, 2023) for the emerging RSV vaccines. Where there is a discrepancy, the broader schedule is reported. Schedule data collected in Q3-Q4 2023.

Table S2. Full list of papers reviewed and included in evidence mapping for country matrices

Title	Main Author	Year	Disease	Included Sample Countries	Value Elements considered	Reference
Effect of Recombinant Zoster Vaccine on Incidence of Herpes Zoster After Autologous Stem Cell Transplantation: A Randomized Clinical Trial	Bastidas	2019	Zoster	France, Germany, Japan	Morbidity	[1]
Influenza vaccine for chronic obstructive pulmonary disease (COPD)	Kopsaftis	2018	Influenza	US, Thailand	Morbidity, Mortality, cost-offsets	[2]
Cost-analysis of opportunistic influenza vaccination in general medical inpatients	Darmaputra	2021	Influenza	Australia	Cost offsets	[3]
Influenza Vaccine Effectiveness Against Influenza-Related Mortality in Australian Hospitalized Patients: A Propensity Score Analysis	Nation	2021	Influenza	Australia	Mortality	[4]
Effectiveness of influenza vaccination in reducing influenza-like illness and related antibiotic prescriptions in adults from a primary care-based case-control study	He	2022	Influenza	Australia	AMR	[5]
Cost-benefit analysis of a national influenza vaccination program in preventing hospitalisation costs in Australian adults aged 50-64 years old	Raj	2019	Influenza	Australia	Morbidity, cost-offsets	[6]

Pneumococcal polysaccharide vaccine is a cost saving strategy for prevention of acute coronary syndrome - PubMed (nih.gov)	Ren	2021	Pneumococcal	Australia	Morbidity, cost-offsets	[7]
Cost-effectiveness of 13-valent pneumococcal conjugate vaccine (PCV13) in older Australians	Dermisropian	2017	Pneumococcal	Australia	Morbidity, Mortality	[8]
Retrospective cost-effectiveness of the 23-valent pneumococcal polysaccharide vaccination program in Australia	Chen	2018	Pneumococcal	Australia	Morbidity, Transmission value, cost-offsets	[9]
Repeated Vaccination Does Not Appear to Impact Upon Influenza Vaccine Effectiveness Against Hospitalization With Confirmed Influenza	Cheng	2017	Influenza	Australia	Morbidity	[10]
A Randomized Trial of Two 2-Dose Influenza Vaccination Strategies for Patients Following Autologous Hematopoietic Stem Cell Transplantation	Teh	2021	Influenza	Australia	Morbidity	[11]
Influenza Vaccination After Myocardial Infarction: A Randomized, Double-Blind, Placebo-Controlled, Multicenter Trial	Fröbert O	2021	Influenza	Australia	Morbidity, Mortality	[12]
Burden, effectiveness and safety of influenza vaccines in elderly, paediatric and pregnant populations	Sullivan	2019	Influenza	Australia, US	Morbidity, Mortality	[13]
Heterogeneity in influenza seasonality and vaccine effectiveness in Australia, Chile, New Zealand and South Africa: early estimates of the 2019 influenza season	Sullivan	2019	Influenza	Australia, South Africa	Morbidity	[14]
The cost-effectiveness of trivalent and quadrivalent influenza vaccination in communities in South Africa, Vietnam and Australia	de Boer	2018	Influenza	Australia, South Africa	Morbidity, Mortality, Transmission value, cost-offsets, patient productivity	[15]
Respiratory Syncytial Virus Prefusion F Protein Vaccine in Older Adults	Papi	2023	RSV	Australia, US, Germany, Brazil, Italy, Japan, South Africa	Morbidity	[16]
The impact of influenza vaccination on morbidity and mortality in the elderly in the major geographic regions of Brazil, 2010 to 2019	Azambuja	2020	Influenza	Brazil	Morbidity, Mortality	[17]
Cost-utility of quadrivalent versus trivalent influenza vaccine in Brazil - comparison of outcomes from different static model types	Van Bellinghen	2018	Influenza	Brazil	Morbidity, Mortality, Cost offsets	[18]
Influenza vaccine effectiveness against influenza A subtypes in Europe: Results from the 2021-2022 I-MOVE primary care multicentre study	Kissling	2022	Influenza	France, Germany	Morbidity	[19]

Cost-effectiveness of public health interventions against human influenza pandemics in France: a methodological contribution from the FLURESP European Commission project	Beresniak	2020	Influenza	France	Transmission value, Cost- offsets	[20]
Health and economic impact of seasonal influenza mass vaccination strategies in European settings: A mathematical modelling and cost-effectiveness analysis	Sandmann	2022	Influenza	France	Morbidity, mortality, transmission value, Cost- offsets	[21]
Invasive pneumococcal disease incidence in children and adults in France during the pneumococcal conjugate vaccine era: an interrupted time-series analysis of data from a 17-year national prospective surveillance study	Ouldali	2021	Pneumococcal	France	Morbidity, transmission value	[22]
The effect of influenza and pneumococcal vaccination in the elderly on health service utilisation and costs: a claims data-based cohort study	Storch	2022	Influenza, pneumococcal	Germany	Cost-offsets	[23]
Cost-effectiveness of the cell-based quadrivalent versus the standard egg-based quadrivalent influenza vaccine in Germany	Cai	2020	Influenza	Germany	Morbidity, Mortality, Cost-offsets, Patient productivity	[24]
Estimating the cost-effectiveness of a sequential pneumococcal vaccination program for adults in Germany	Kuchenbecker	2018	Pneumococcal	Germany	Morbidity, Mortality, Transmission value, Cost-offsets, Patient productivity	[25]
Long-term efficacy data for the recombinant zoster vaccine: impact on public health and cost effectiveness in Germany	Curran	2021	Zoster	Germany	Morbidity, Mortality, Cost-offsets	[26]
Cost-effectiveness of the recombinant zoster vaccine in the German population aged ≥60 years old	Oorschot	2019	Zoster	Germany	Morbidity, Mortality, Cost-offsets	[27]
Pneumococcal vaccination in older persons: where are we today?	Van Buynder	2018	Pneumococcal	Australia, US, Japan, Spain, Germany	Morbidity, Cost-offsets	[28]
A systematic review of the health economic consequences of quadrivalent influenza vaccination	de Boer	2017	Influenza	US, Austraila, Germany	Morbidity, Mortality, Cost-offsets, patient productivity	[29]
Impact of influenza syndrome and flu vaccine on survival of cancer patients during immunotherapy in the INVIDIa study	Bersanelli	2020	Influenza	Italy	Morbidity, Mortality	[30]
Effectiveness of influenza vaccine in reducing influenza-associated hospitalizations and deaths	Fabiani	2020	Influenza	Italy	Morbidity, Mortality	[31]

among the elderly population; Lazio region, Italy, season 2016-2017						
Influenza vaccination and absenteeism among healthy working adults: a cost-benefit analysis	Ferro	2020	Influenza	Italy	Cost-offsets, patient productivity	[32]
Estimating the fiscal impact of three vaccination strategies in Italy	Ruggeri	2020	Influenza, pneumococcal, zoster	Italy	Cost-offsets, patient productivity	[33]
Brand-specific influenza vaccine effectiveness estimates during 2019/20 season in Europe - Results from the DRIVE EU study platform	Stuurman	2021	Influenza	Italy, France	Morbidity	[34]
The potential public health impact of Herpes Zoster vaccination in the 65 years of age cohort in Italy	Volpi	2019	Zoster	Italy	Morbidity, Mortality, Cost-offsets, patient productivity	[35]
Cost-effectiveness analysis of influenza vaccination during pregnancy in Japan	Hoshi	2020	Influenza	Japan	Cost-offsets	[36]
Effectiveness and cost-effectiveness of influenza vaccination for elderly people	Sugishita and Sugawara	2021	Influenza	Japan	Morbidity, Cost-offsets	[37]
Simulation studies to assess the long-term effects of Japan's change from trivalent to quadrivalent influenza vaccination	Tzuzuki	2018	Influenza	Japan	Transmission value, Cost-offsets, patient productivity	[38]
Influenza vaccine effectiveness against hospitalization during the 2018/2019 season among older persons aged ≥ 75 years in Japan: The LIFE-VENUS Study	Mimura	2022	Influenza	Japan	Morbidity	[39]
Preventive effects of pneumococcal and influenza vaccines on community-acquired pneumonia in older individuals in Japan: a case-control study	Suzuki	2021	Influenza, pneumococcal	Japan	Morbidity	[40]
Influenza vaccine showed a good preventive effect against influenza-associated hospitalization among elderly patients, during the 2016/17 season in Japan	Seki	2018	Influenza	Japan	Morbidity	[41]
Effectiveness of inactivated influenza vaccine against laboratory-confirmed influenza pneumonia among adults aged ≥65 years in Japan	Suzuki	2018	Influenza	Japan	Morbidity, Mortality	[42]
Cost-effectiveness analyses of 15- and 20-valent pneumococcal conjugate vaccines for Japanese elderly	Hoshi	2022	Pneumococcal	Japan	Cost-offsets	[43]
Cost-effectiveness analysis for PCV13 in adults 60 years and over with underlying medical conditions which put them at an elevated risk of pneumococcal disease in Japan	Igarashi	2021	Pneumococcal	Japan	Morbidity, Mortality, Cost-offsets, patient	[44]

					productivity, carer productivity	
A cost-effectiveness analysis of revaccination and catch-up strategies with the 23-valent pneumococcal polysaccharide vaccine (PPV23) in older adults in Japan	Jiang	2018	Pneumococcal	Japan	Morbidity, Mortality, Cost offsets	[45]
Cost-Effectiveness Analysis Update of the Adjuvanted Recombinant Zoster Vaccine in Japanese Older Adults	Teng	2022	Zoster	Japan	Morbidity, Mortality, Cost-offsets, patient productivity, carer productivity	[46]
Cost-effectiveness of varicella vaccine against herpes zoster and post-herpetic neuralgia for elderly in Japan	Hoshi	2017	Zoster	Japan	Cost offsets	[47]
Cost-effectiveness of Recombinant Zoster Vaccine (RZV) and Varicella Vaccine Live (VVL) against herpes zoster and post-herpetic neuralgia among adults aged 65 and over in Japan	Hoshi	2019	Zoster	Japan	Cost offsets	[48]
The adjuvanted recombinant zoster vaccine is efficacious and safe in Asian adults ≥ 50 years of age: a sub-cohort analysis of the ZOE-50 and ZOE-70 randomized trials	Kim	2021	Zoster	Japan	Morbidity	[49]
Pneumococcal vaccination reduces in-hospital mortality, length of stay and medical expenditure in hospitalized elderly patients	Naito	2020	Pneumococcal	Japan	Morbidity, Mortality, Cost offsets	[50]
Interim 2019/2020 Influenza Vaccine Effectiveness in Japan from October 2019 to January 2020	Tsuzuki	2021	Influenza	Japan	Morbidity	[51]
Public health impact and economic benefits of quadrivalent influenza vaccine in Latin America	Jamotte	2017	Influenza	Brazil	Morbidity, Mortality, Cost-offsets	[52]
Seasonal influenza vaccine effectiveness against laboratory-confirmed influenza hospitalizations - Latin America, 2013	El Omeiri	2017	Influenza	Brazil	Morbidity	[53]
Prevention of influenza during mismatched seasons in older adults with an MF59-adjuvanted quadrivalent influenza vaccine: a randomised, controlled, multicentre, phase 3 efficacy study	Beran	2021	Influenza	Poland, Thailand	Morbidity, Mortality	[54]
How large could the public health impact of introducing recombinant zoster vaccination for people aged ≥50 years in five Latin American countries be?	Han	2023	Zoster	Brazil	Morbidity	[55]
A cost-effectiveness analysis of South Africa's seasonal influenza vaccination programme	Edoka	2021	Influenza	South Africa	Morbidity, Mortality, Cost-offsets, patient productivity, carer productivity	[56]

Prioritization of risk groups for influenza vaccination in resource limited settings - A case study from South Africa	McMorrow	2019	Influenza	South Africa	Morbidity, Mortality, Cost-offsets	[57]
The cost-effectiveness of using pneumococcal conjugate vaccine (PCV13) versus pneumococcal polysaccharide vaccine (PPSV23), in South African adults	Feldman	2020	Pneumococcal	South Africa	Morbidity, Mortality, Cost-offsets, patient productivity	[58]
A review of the cost-effectiveness of adult influenza vaccination and other preventive services	Dabestani	2019	Influenza	US	Morbidity, Mortality, Cost-offsets	[59]
Potential Cost-Effectiveness of a Universal Influenza Vaccine in Older Adults	France	2018	Influenza	US	Morbidity, Mortality, Cost-offsets, patient productivity, carer productivity	[60]
Does Choice of Influenza Vaccine Type Change Disease Burden and Cost-Effectiveness in the United States? An Agent-Based Modeling Study	DePasse	2017	Influenza	US	Morbidity, Mortality, transmission value, Cost-offsets, patient productivity	[61]
Cost-effectiveness of influenza vaccination during pregnancy	Chaiken	2021	Influenza	US	Morbidity, Mortality, Cost-offsets	[62]
Effectiveness of Trivalent and Quadrivalent Inactivated Vaccines Against Influenza B in the United States, 2011-2012 to 2016-2017	Gaglani	2021	Influenza	US	Morbidity	[63]
Cost-effectiveness and public health impact of alternative influenza vaccination strategies in high-risk adults	Raviotta	2017	Influenza	US	Morbidity, Mortality, Cost-offsets, patient productivity	[64]
Cost-effectiveness of Pneumococcal Vaccination Among Patients With CKD in the United States	Ishigami	2019	Pneumococcal	US	Morbidity, Mortality, Cost-offsets	[65]
Higher-Valency Pneumococcal Conjugate Vaccines: An Exploratory Cost-Effectiveness Analysis in U.S. Seniors	Smith	2021	Pneumococcal	US	Morbidity, Mortality, Cost-offsets	[66]
Cost-effectiveness of implementing 13-valent pneumococcal conjugate vaccine for U.S. adults aged 19 years and older with underlying conditions	Kobayashi	2021	Pneumococcal	US	Morbidity, Mortality, Cost-offsets	[67]
Cost-Effectiveness of Pneumococcal Vaccination Policies and Uptake Programs in US Older Populations	Wateska	2020	Pneumococcal	US	Morbidity, Mortality, Cost-offsets	[68]
Cost-effectiveness of adult pneumococcal vaccination policies in underserved minorities aged	Wateska	2019	Pneumococcal	US	Morbidity, Mortality,	[69]

50-64 years compared to the US general population					Cost-offsets, Social equity	
Cost-Effectiveness of Pneumococcal Vaccination and Uptake Improvement Programs in Underserved and General Population Adults Aged	Wateska	2020	Pneumococcal	US	Morbidity, Mortality, Cost-offsets, Social equity	[70]
Cost-effectiveness of an adjuvanted recombinant zoster vaccine in older adults in the United States who have been previously vaccinated with zoster vaccine live	Curran	2019	Zoster	US	Morbidity, Mortality, Cost-offsets, patient productivity	[71]
Cost-effectiveness of an Adjuvanted Recombinant Zoster Vaccine in older adults in the United States	Curran	2018	Zoster	US	Morbidity, Mortality, Cost-offsets, patient productivity	[72]
Cost-effectiveness of herpes zoster vaccines in the U.S.: A systematic review	Meredith	2022	Zoster	US	Morbidity, Mortality, cost-offsets	[73]
A Cost-effectiveness Analysis of an Adjuvanted Subunit Vaccine for the Prevention of Herpes Zoster and Post-herpetic Neuralgia	Carpenter	2019	Zoster	US	Morbidity, Mortality, Cost-offsets, patient productivity	[74]
Cost Effectiveness of a Shingles Vaccine Booster for Currently Vaccinated Adults in the U.S	Le	2017	Zoster	US	Morbidity, Cost-offsets, patient productivity	[75]
Cost-effectiveness of the Recommendations of the Advisory Committee on Immunization Practices for the Recombinant Adjuvanted Zoster Subunit Vaccine	Le	2018	Zoster	US	Morbidity, Cost-offsets, patient productivity	[76]
Comparative Effectiveness of High-Dose Versus Standard-Dose Influenza Vaccine Among Patients Receiving Maintenance Hemodialysis	Butler	2019	Influenza	US	Morbidity, Mortality	[77]
Comparison of vaccine effectiveness against influenza hospitalization of cell-based and egg-based influenza vaccines, 2017-2018	Bruxvoort	2019	Influenza	US	Morbidity	[78]
Comparative effectiveness of high-dose versus standard-dose influenza vaccination on numbers of US nursing home residents admitted to hospital: a cluster-randomised trial	Gravenstein	2017	Influenza	US	Morbidity	[79]
Relative Effectiveness of Cell-Cultured and Egg-Based Influenza Vaccines Among Elderly Persons in the United States, 2017-2018	Izurieta	2019	Influenza	US	Morbidity	[80]
Relative Effectiveness of Influenza Vaccines Among the United States Elderly, 2018-2019	Izurieta	2020	Influenza	US	Morbidity	[81]

Recombinant Zoster Vaccine (Shingrix): Real-World Effectiveness in the First 2 Years Post-Licensure	Izurieta	2021	Zoster	US	Morbidity	[82]
Effectiveness of 13-Valent Pneumococcal Conjugate Vaccine Against Hospitalization for Community-Acquired Pneumonia in Older US Adults: A Test-Negative Design	McLaughlin	2018	Pneumococcal	US	Morbidity	[83]
Pneumococcal Vaccination in Adults Aged ≥65 Years: Cost-Effectiveness and Health Impact in U.S. Populations	Wateska	2021	Pneumococcal	US	Morbidity, Mortality, Cost-offsets	[84]
Modeling Respiratory Syncytial Virus Adult Vaccination in the United States with a Dynamic Transmission Model	Effelterre	2023	RSV	US	Morbidity, Mortality, Transmission	[85]
Clinical and economic outcomes associated with respiratory syncytial virus vaccination in older adults in the United States	Herring	2022	RSV	US	Morbidity, Mortality, Cost-offsets	[86]
Effect of High-Dose Trivalent vs Standard-Dose Quadrivalent Influenza Vaccine on Mortality or Cardiopulmonary Hospitalization in Patients With High-risk Cardiovascular Disease: A Randomized Clinical Trial	Vardeny	2021	Influenza	US	Morbidty, Mortality	[87]
Economic evaluation of high-dose inactivated influenza vaccine in adults aged ≥65 years: A systematic literature review	Colrat	2021	Influenza	US	Morbidity, Mortality, Cost-offsets, patient productivity	[88]
Efficacy and Safety of a Bivalent RSV Prefusion F Vaccine in Older Adults	Walsh	2023	RSV	US, Japan, South Africa	Morbidity	[89]
High-dose influenza vaccines for the prevention of hospitalization due to cardiovascular events in older adults in the nursing home: Post-hoc analysis of a cluster-randomized trial	Saade	2022	Influenza	USA	Morbidity	[90]
Safety, Reactogenicity, and Health-Related Quality of Life After Trivalent Adjuvanted vs Trivalent High-Dose Inactivated Influenza Vaccines in Older Adults: A Randomized Clinical Trial	Schmader	2021	Influenza	USA	Morbidity	[91]
Influenza vaccine effectiveness among patients with high-risk medical conditions in the United States, 2012-2016	Shang	2018	Influenza	USA	Morbidity	[92]
Comparative Effectiveness of High-Dose Versus Standard-Dose Influenza Vaccines Among US Medicare Beneficiaries in Preventing Postinfluenza Deaths During 2012-2013 and 2013-2014	Shay	2017	Influenza	USA	Morbidity, Mortality	[93]
Cost-Effectiveness of the 4 Pillars Practice Transformation Program to Improve Vaccination of Adults Aged 65 and Older	Smith	2017	Pneumococcal, influenza	USA	Morbidity, Mortality, Cost-offsets	[94]

Burden of pneumococcal disease among adults in Southern Europe (Spain, Portugal, Italy, and Greece): a systematic review and meta-analysis	Navarro-Torné	2021	Pneumo-coccal	Italy	Morbidity	[95]

References

[1] Bastidas, A.; de la Serna, J.; El Idrissi, M.; Oostvogels, L.; Quittet, P.; López-Jiménez, J.; Vural, F.; Pohlreich, D.; Zuckerman, T.; Issa, N. C.; et al. Effect of Recombinant Zoster Vaccine on Incidence of Herpes Zoster After Autologous Stem Cell Transplantation: A Randomized Clinical Trial. *JAMA*, **2019**, *322* (2), 123–133. https://doi.org/10.1001/jama.2019.9053.

[2] Kopsaftis, Z.; Wood-Baker, R.; Poole, P. Influenza Vaccine for Chronic Obstructive Pulmonary Disease (COPD). *Cochrane Database Syst. Rev.*, **2018**, *2018* (6), CD002733. https://doi.org/10.1002/14651858.CD002733.pub3.

[3] Darmaputra, D. C.; Zaman, F. Y.; Khu, Y. L.; Nagalingam, V.; Liew, D.; Aung, A. K. Cost-Analysis of Opportunistic Influenza Vaccination in General Medical Inpatients. *Intern. Med. J.*, **2021**, *51* (4), 591–595. https://doi.org/10.1111/imj.15270.

[4] Monica L. Nation,1,2, Robert Moss,2, Matthew J. Spittal,2; Tom Kotsimbos,3; Paul M. Kelly,4; and Allen C. Cheng5. Influenza Vaccine Effectiveness Against Influenza-Related Mortality in Australian Hospitalized Patients: A Propensity Score Analysis, 2021.

[5] He, W.-Q.; Gianacas, C.; Muscatello, D. J.; Newall, A. T.; McIntyre, P.; Cheng, A. C.; Liu, B. Effectiveness of Influenza Vaccination in Reducing Influenza-like Illness and Related Antibiotic Prescriptions in Adults from a Primary Care-Based Case-Control Study. *J. Infect.*, **2022**, *85* (6), 660–665. https://doi.org/10.1016/j.jinf.2022.10.028.

[6] Raj, S. M.; Chughtai, A. A.; Sharma, A.; Tan, T. C.; MacIntyre, C. R. Cost-Benefit Analysis of a National Influenza Vaccination Program in Preventing Hospitalisation Costs in Australian Adults Aged 50–64 Years Old. *Vaccine*, **2019**, *37* (40), 5979–5985. https://doi.org/10.1016/j.vaccine.2019.08.028.

[7] Ren, S.; Attia, J.; Li, S. C.; Newby, D. Pneumococcal Polysaccharide Vaccine Is a Cost Saving Strategy for Prevention of Acute Coronary Syndrome. *Vaccine*, **2021**, *39* (12), 1721–1726. https://doi.org/10.1016/j.vaccine.2021.02.019.

[8] Cho, B.-H.; Stoecker, C.; Link-Gelles, R.; Moore, M. R. Cost-Effectiveness of Administering 13-Valent Pneumococcal Conjugate Vaccine in Addition to 23-Valent Pneumococcal Polysaccharide Vaccine to Adults with Immunocompromising Conditions. *Vaccine*, **2013**, *31* (50), 6011–6021. https://doi.org/10.1016/j.vaccine.2013.10.024.

[9] C. Chen a, P. Beutels b, J. Wood a, R. Menzies a, C.R. MacIntyre a, P. McIntyre c, A.T. Newall. Retrospective Cost-Effectiveness of the 23-Valent Pneumococcal Polysaccharide Vaccination Program in Australia. **2018**.

[10] Allen C. Cheng,; Kristine K. Macartney,; Grant W. Waterer,; Tom Kotsimbos,; Paul M. Kelly,; Christopher C. Blyth. Repeated Vaccination Does Not Appear to Impact Upon Influenza Vaccine Effectiveness Against Hospitalization With Confirmed Influenza. **2017**.

[11] Teh, B. W.; Leung, V. K. Y.; Mordant, F. L.; Sullivan, S. G.; Joyce, T.; Harrison, S. J.; Khvorov, A.; Barr, I. G.; Subbarao, K.; Slavin, M. A.; et al. A Randomized Trial of Two 2-Dose Influenza Vaccination Strategies for Patients Following Autologous Hematopoietic Stem Cell Transplantation. *Clin. Infect. Dis. Off. Publ. Infect. Dis. Soc. Am.*, **2021**, *73* (11), e4269–e4277. https://doi.org/10.1093/cid/ciaa1711.

[12] Ole Fröbert, MD1; Matthias Götberg, MD2; David Erlinge, MD2; Zubair Akhtar, MPH3;Evald H. Christiansen, MD4; Chandini R. MacIntyre, MBBS, PhD5;Keith G. Oldroyd, MBChB, MD6; Zuzana Motovska, MD7; Andrejs Erglis, MD8;Rasmus Moer, MD9; Ota Hlinomaz, MD10; Lars Jakobsen, MD4; Thomas Engstrøm, MD11;Lisette O. Jensen, MD12; Christian O. Fallesen, MD12; Svend E. Jensen, MD13;Oskar Angerås, MD14; Fredrik Calais, MD1;

Amra Kåregren, MD15; Jörg Lauermann, MD16;Arash Mokhtari, MD2; Johan Nilsson, MD17; Jonas Persson, MD18; Per Stalby, MD19;Abu K.M.M. Islam, MD20; Afzalur Rahman, MD20; Fazila Malik, MBBS21;Sohel Choudhury, PhD21; Timothy Collier, MSc22; Stuart J. Pocock, PhD22; John Pernow, M. Influenza Vaccination After Myocardial Infarction: A Randomized, Double-Blind, Placebo-Controlled, Multicenter Trial. **2021**.

[13] Sullivan, S. G.; Price, O. H.; Regan, A. K. Burden, Effectiveness and Safety of Influenza Vaccines in Elderly, Paediatric and Pregnant Populations. *Ther. Adv. Vaccines Immunother.*, **2019**, *7*, 2515135519826481. https://doi.org/10.1177/2515135519826481.

[14] Sheena G Sullivan, 1 Carmen S Arriola, 2 Judy Bocacao, 3 Pamela Burgos, 4 Patricia Bustos, 5 Kylie S Carville, 6 Allen C Cheng, 7 , 8 Monique BM Chilver, 9 Cheryl Cohen, 10 Yi-Mo Deng, 11 Nathalie El Omeiri, 12 Rodrigo A Fasce, 13 Orienka Hellferscee, 10 Q Sue Huang, 3 Cecilia Gonzalez, 4 Lauren Jelley, 3 Vivian KY Leung, 1 Liza Lopez, 14 Johanna M McAnerney, 10 Andrea McNeill, 14 Maria F Olivares, 15 Heidi Peck, 11 Viviana Sotomayor, 15 Stefano Tempia, 2 , 10 , 16 , 17 Natalia Vergara, 15 Anne von Gottberg, 10 Sibongile Walaza, 10 and Timothy Wood 14. Heterogeneity in Influenza Seasonality and Vaccine Effectiveness in Australia, Chile, New Zealand and South Africa: Early Estimates of the 2019 Influenza Season. **2019**.

[15] de Boer, P. T.; Kelso, J. K.; Halder, N.; Nguyen, T.-P.-L.; Moyes, J.; Cohen, C.; Barr, I. G.; Postma, M. J.; Milne, G. J. The Cost-Effectiveness of Trivalent and Quadrivalent Influenza Vaccination in Communities in South Africa, Vietnam and Australia. *Vaccine*, **2018**, *36* (7), 997–1007. https://doi.org/10.1016/j.vaccine.2017.12.073.

[16] Papi, A.; Ison, M. G.; Langley, J. M.; Lee, D.-G.; Leroux-Roels, I.; Martinon-Torres, F.; Schwarz, T. F.; van Zyl-Smit, R. N.; Campora, L.; Dezutter, N.; et al. Respiratory Syncytial Virus Prefusion F Protein Vaccine in Older Adults. *N. Engl. J. Med.*, **2023**, *388* (7), 595–608. https://doi.org/10.1056/NEJMoa2209604.

[17] Azambuja, H. C. S.; Carrijo, M. F.; Martins, T. C. R.; Luchesi, B. M. The Impact of Influenza Vaccination on Morbidity and Mortality in the Elderly in the Major Geographic Regions of Brazil, 2010 to 2019. *Cad. Saúde Pública*, **2020**, *36*, e00040120.

[18] Van Bellinghen, L.-A.; Marijam, A.; Tannus Branco de Araujo, G.; Gomez, J.; Van Vlaenderen, I. Cost-Utility of Quadrivalent versus Trivalent Influenza Vaccine in Brazil – Comparison of Outcomes from Different Static Model Types. *Braz. J. Infect. Dis.*, **2018**, *22* (1), 1–10. https://doi.org/10.1016/j.bjid.2017.11.004.

[19] Kissling, E.; Pozo, F.; Martínez-Baz, I.; Buda, S.; Vilcu, A.; Domegan, L.; Mazagatos, C.; Dijkstra, F.; Latorre-Margalef, N.; Kurečić Filipović, S.; et al. Influenza Vaccine Effectiveness against Influenza A Subtypes in Europe: Results from the 2021–2022 I-MOVE Primary Care Multicentre Study. *Influenza Other Respir. Viruses*, **2022**, *17* (1), e13069. https://doi.org/10.1111/irv.13069.

[20] Beresniak, A.; Rizzo, C.; Oxford, J.; Goryński, P.; Pistol, A.; Fabiani, M.; Napoli, C.; Barral, M.; Niddam, L.; Bounekkar, A.; et al. Cost-Effectiveness of Public Health Interventions against Human Influenza Pandemics in France: A Methodological Contribution from the FLURESP European Commission Project. *Eur. J. Public Health*, **2019**, ckz074. https://doi.org/10.1093/eurpub/ckz074.

[21] Sandmann, F. G.; van Leeuwen, E.; Bernard-Stoecklin, S.; Casado, I.; Castilla, J.; Domegan, L.; Gherasim, A.; Hooiveld, M.; Kislaya, I.; Larrauri, A.; et al. Health and Economic Impact of Seasonal Influenza Mass Vaccination Strategies in European Settings: A Mathematical Modelling and Cost-Effectiveness Analysis. *Vaccine*, **2022**, *40* (9), 1306–1315. https://doi.org/10.1016/j.vaccine.2022.01.015.

[22] Naïm Ouldali , Emmanuelle Varon 2, Corinne Levy 3, François Angoulvant 4, Scarlett Georges 5, Marie-Cécile Ploy 6, Marie Kempf 7, Julie Cremniter 8, Robert Cohen 9, Daniel Levy Bruhl 5, Kostas Danis 5. Invasive Pneumococcal Disease Incidence in Children and Adults in France during the Pneumococcal Conjugate Vaccine

Era: An Inter-Rupted Time-Series Analysis of Data from a 17-Year National Prospective Surveillance Study. *HttpswwwthelancetcomjournalslaninfarticlePIIS1473-30992030165-1abstract*.

[23] Storch, J.; Fleischmann-Struzek, C.; Rose, N.; Lehmann, T.; Mikolajetz, A.; Maddela, S.; Pletz, M. W.; Forstner, C.; Wichmann, O.; Neufeind, J.; et al. The Effect of Influenza and Pneumococcal Vaccination in the Elderly on Health Service Utilisation and Costs: A Claims Data-Based Cohort Study. *Eur. J. Health Econ.*, **2022**, *23* (1), 67–80. https://doi.org/10.1007/s10198-021-01343-8.

[24] Cai, R.; Gerlier, L.; Eichner, M.; Schwehm, M.; Rajaram, S.; Mould-Quevedo, J.; Lamotte, M. Cost-Effectiveness of the Cell-Based Quadrivalent versus the Standard Egg-Based Quadrivalent Influenza Vaccine in Germany. *J. Med. Econ.*, **2021**, *24* (1), 490–501. https://doi.org/10.1080/13696998.2021.1908000.

[25] Kuchenbecker, U.; Chase, D.; Reichert, A.; Schiffner-Rohe, J.; Atwood, M. Estimating the Cost-Effectiveness of a Sequential Pneumococcal Vaccination Program for Adults in Germany. *PLoS ONE*, **2018**, *13* (5). https://doi.org/10.1371/journal.pone.0197905.

[26] Curran, D.; Van Oorschot, D.; Matthews, S.; Hain, J.; Salem, A. E.; Schwarz, M. Long-Term Efficacy Data for the Recombinant Zoster Vaccine: Impact on Public Health and Cost Effectiveness in Germany. *Hum. Vaccines Immunother.*, *17* (12), 5296–5303. https://doi.org/10.1080/21645515.2021.2002085.

[27] Van Oorschot, D.; Anastassopoulou, A.; Poulsen Nautrup, B.; Varghese, L.; von Krempelhuber, A.; Neine, M.; Lorenc, S.; Curran, D. Cost-Effectiveness of the Recombinant Zoster Vaccine in the German Population Aged ≥60 Years Old. *Hum. Vaccines Immunother.*, **2019**, *15* (1), 34–44. https://doi.org/10.1080/21645515.2018.1509645.

[28] Van Buynder, P.; Booy, R. Pneumococcal Vaccination in Older Persons: Where Are We Today? *Pneumonia*, **2018**, *10*, 1. https://doi.org/10.1186/s41479-017-0045-y.

[29] de Boer, P. T.; van Maanen, B. M.; Damm, O.; Ultsch, B.; Dolk, F. C. K.; Crépey, P.; Pitman, R.; Wilschut, J. C.; Postma, M. J. A Systematic Review of the Health Economic Consequences of Quadrivalent Influenza Vaccination. *Expert Rev. Pharmacoecon. Outcomes Res.*, **2017**, *17* (3), 249–265. https://doi.org/10.1080/14737167.2017.1343145.

[30] Melissa Bersanelli , Sebastiano Buti, Giuseppe Luigi Banna , Ugo De Giorgi, Alessio Cortellini, Sara Elena Rebuzzi, Marcello Tiseo, Giuseppe Fornarini, Francesca Mazzoni, Stefano Panni, Michele De Tursi, Pietro Di Marino, Sabrina Rossetti, Ernesto Rossi, Silverio Tomao, Emmanuele De Luca, Mariella Sorarù, Claudia Mucciarini, Francesco Atzori, Leonardo La Torre, Maria Giuseppa Vitale, Valentino Martelli, Pierangela Sepe, Veronica Mollica, Vanja Vaccaro, Giovanni Schinzari, Corrado Ficorella, Francesco Massari, Antonio Maestri, Roberto Sabbatini, Teodoro Sava, Massimo Di Maio, Elena Verzoni, Giuseppe Procopio & Diana Giannarelli. Impact of Influenza Syndrome and Flu Vaccine on Survival of Cancer Patients during Immunotherapy in the INVIDIa Study. **2020**.

[31] Massimo Fabiani 1, Enrico Volpe 2, Maurizio Faraone 2, Antonino Bella 1, Patrizio Pezzotti 1, Francesco Chini 2. Effectiveness of Influenza Vaccine in Reducing Influenza-Associated Hospitalizations and Deaths among the Elderly Population; Lazio Region, Italy, Season 2016-2017. **2020**.

[32] Ferro, A.; Bordin, P.; Benacchio, L. Influenza Vaccination and Absenteeism among Healthy Working Adults: A Cost-Benefit Analysis. *Ann. Ig. Med. Prev. E Comunità*, **2020**, No. 3, 234–244. https://doi.org/10.7416/ai.2020.2346.

[33] Ruggeri, M.; Di Brino, E.; Cicchetti, A. Estimating the Fiscal Impact of Three Vaccination Strategies in Italy. *Int. J. Technol. Assess. Health Care*, **2020**, *36* (2), 133–138. https://doi.org/10.1017/S0266462320000069.

[34] Anke L Stuurman 1, Jorne Biccler 2, Antonio Carmona 3, Alexandre Descamps 4, Javier Díez-Domingo 5, Cintia Muñoz Quiles 6, Hanna Nohynek 7, Caterina Rizzo 8, Margarita Riera-Montes 9; Brand-Specific Influenza Vaccine Effectiveness Estimates during 2019/20 Season in Europe - Results from the DRIVE EU Study Platform. **2021**.

[35] Volpi, A.; Boccalini, S.; Dari, S.; Clarke, C.; Curran, D.; Loiacono, I.; Pitrelli, A.; Puggina, A.; Tosatto, R.; Van Oorschot, D.; et al. The Potential Public Health Impact of Herpes Zoster Vaccination in the 65 Years of Age Cohort in Italy. *Hum. Vaccines Immunother.*, **2019**, *16* (2), 327–334. https://doi.org/10.1080/21645515.2019.1657753.

[36] Hoshi, S.; Shono, A.; Seposo, X.; Okubo, I.; Kondo, M. Cost-Effectiveness Analysis of Influenza Vaccination during Pregnancy in Japan. *Vaccine*, **2020**, *38* (46), 7363–7371. https://doi.org/10.1016/j.vaccine.2020.09.024.

[37] Sugishita, Y.; Sugawara, T. Effectiveness and Cost-Effectiveness of Influenza Vaccination for Elderly People. *Vaccine*, **2021**, *39* (52), 7531–7540. https://doi.org/10.1016/j.vaccine.2021.09.054.

[38] Tsuzuki, S.; Schwehm, M.; Eichner, M. Simulation Studies to Assess the Long-Term Effects of Japan's Change from Trivalent to Quadrivalent Influenza Vaccination. *Vaccine*, **2018**, *36* (5), 624–630. https://doi.org/10.1016/j.vaccine.2017.12.058.

[39] Mimura, W.; Ishiguro, C.; Fukuda, H. Influenza Vaccine Effectiveness against Hospitalization during the 2018/2019 Season among Older Persons Aged ≥ 75 years in Japan: The LIFE-VENUS Study. *Vaccine*, **2022**, *40* (34), 5023–5029. https://doi.org/10.1016/j.vaccine.2022.07.002.

[40] Kanzo Suzukia,b, Kyoko Kondoc; , Masakazu Washiod; , Kei Nakashima e; , Sakae Kanf; , Seiichiro Imaig,h,; Kunihiko Yoshimurai; , Chiharu Otaj; , Satoko Ohfujik,l, Wakaba Fukushimak,l, and Yoshio Hirotak,m,n and the Study Group; for Pneumonia in the Elderly Individuals#. Preventive Effects of Pneumococcal and Influenza Vaccines on Community-Acquired Pneumonia in Older Individuals in Japan: A Case-Control Study. **2019**.

[41] Seki, Y.; Onose, A.; Murayama, T.; Koide, C.; Sugaya, N. Influenza Vaccine Showed a Good Preventive Effect against Influenza-Associated Hospitalization among Elderly Patients, during the 2016/17 Season in Japan. *J. Infect. Chemother.*, **2018**, *24* (11), 873–880. https://doi.org/10.1016/j.jiac.2018.07.013.

[42] Suzuki, M.; Katsurada, N.; Le, M. N.; Kaneko, N.; Yaegashi, M.; Hosokawa, N.; Otsuka, Y.; Aoshima, M.; Yoshida, L. M.; Morimoto, K. Effectiveness of Inactivated Influenza Vaccine against Laboratory-Confirmed Influenza Pneumonia among Adults Aged ≥65 years in Japan. *Vaccine*, **2018**, *36* (21), 2960–2967. https://doi.org/10.1016/j.vaccine.2018.04.037.

[43] Shu-ling Hoshi a, Aiko Shono b, Xerxes Seposo c, Reiko Okubo a d, Masahide Kondo a; a; Department of Health Care Policy and Health Economics, Faculty of Medicine, University of Tsukuba, 1-1-1, Tennoudai, Tsukuba, Ibaraki 3058577, Japan; b; Laboratory of Social Pharmacy and Regulatory Science, Showa Pharmaceutical University, Machida, Tokyo 194-8543, Japan; c; Department of Hygiene, Graduate School of Medicine, Hokkaido University, Kita 15, Nishi 7, Kita-ku, Sapporo 060-8638, Japan; d; Department of Clinical Laboratory Medicine, University of Tsukuba Hospital, Tsukuba, Ibaraki, Japan. Cost-Effectiveness Analyses of 15- and 20-Valent Pneumococcal Conjugate Vaccines for Japanese Elderly. **2022**.

[44] Igarashi, A.; Hirose, E.; Kobayashi, Y.; Yonemoto, N.; Lee, B. Cost-Effectiveness Analysis for PCV13 in Adults 60 Years and over with Underlying Medical Conditions Which Put Them at an Elevated Risk of Pneumococcal Disease in Japan. *Expert Rev. Vaccines*, **2021**, *20* (9), 1153–1165. https://doi.org/10.1080/14760584.2021.1952869.

[45] Jiang, Y.; Yang, X.; Taniguchi, K.; Petigara, T.; Abe, M. A Cost-Effectiveness Analysis of Revaccination and Catch-up Strategies with the 23-Valent Pneumococcal Polysaccharide Vaccine (PPV23) in Older Adults in Japan. *J. Med. Econ.*, **2018**, *21* (7), 687–697. https://doi.org/10.1080/13696998.2018.1465272.

[46] Teng, L.; Mizukami, A.; Ng, C.; Giannelos, N.; Curran, D.; Sato, T.; Lee, C.; Matsuki, T. Cost-Effectiveness Analysis Update of the Adjuvanted Recombinant Zoster Vaccine in Japanese Older Adults. *Dermatol. Ther.*, **2022**, *12* (6), 1447–1467. https://doi.org/10.1007/s13555-022-00744-8.

[47] Shu-ling Hoshi, Masahide Kondo, Ichiro Okubo; Department of Health Care Policy and Health Economics, Faculty of Medicine, University of Tsukuba, 1-1-1, Tennoudai, Tsukuba, Ibaraki 3058577, Japan. Cost-Effectiveness of Varicella Vaccine against Herpes Zoster and Post-Herpetic Neuralgia for Elderly in Japan. **2017**.

[48] Cost-Effectiveness of Recombinant Zoster Vaccine (RZV) and Varicella Vaccine Live (VVL) against Herpes Zoster and Post-Herpetic Neuralgia among Adults Aged 65 and over in Japan.

[49] Joon Hyung Kim a; , John Diaz-Decaro a; , Ning Jiangb; , Shinn-Jang Hwang c,d, Eun Ju Chooe; , Maribel Cof; ,; Andrew Hastiea; , David Shu Cheong Huig; , Junya Irimajirih; , Jacob Leei; et al. The Adjuvanted Recombinant Zoster Vaccine Is Efficacious and Safe in Asian Adults ≥ 50 Years of Age: A Sub-Cohort Analysis of the ZOE-50 and ZOE-70 Randomized Trials. **2021**.

[50] Naito, T.; Suzuki, M.; Kanazawa, A.; Takahashi, H.; Fujibayashi, K.; Yokokawa, H.; Kuwatsuru, R.; Watanabe, A. Pneumococcal Vaccination Reduces In-Hospital Mortality, Length of Stay and Medical Expenditure in Hospitalized Elderly Patients. *J. Infect. Chemother.*, **2020**, *26* (7), 715–721. https://doi.org/10.1016/j.jiac.2020.03.016.

[51] Tsuzuki, S.; Ishikane, M.; Matsunaga, N.; Morioka, S.; Yu, J.; Inagaki, T.; Yamamoto, M.; Ohmagari, N. Interim 2019/2020 Influenza Vaccine Effectiveness in Japan from October 2019 to January 2020. *Jpn. J. Infect. Dis.*, **2021**, *74* (3), 175–179. https://doi.org/10.7883/yoken.JJID.2020.177.

[52] Jamotte, A.; Clay, E.; Macabeo, B.; Caicedo, A.; Lopez, J. G.; Bricks, L.; Prada, M. R.; Marrugo, R.; Alfonso, P.; Arévalo, B. M.; et al. Public Health Impact and Economic Benefits of Quadrivalent Influenza Vaccine in Latin America. *Hum. Vaccines Immunother.*, **2017**.

[53] Nathalie El Omeiri,a,b,⁎ Eduardo Azziz-Baumgartner,c Mark G. Thompson,c the REVELAC-i network participants,1 Wilfrido Clará,d Mauricio Cerpa,e Rakhee Palekar,e Sara Mirza,c and Alba María Ropero-Álvareza. Seasonal Influenza Vaccine Effectiveness against Laboratory-Confirmed Influenza Hospitalizations – Latin America, 2013. **2018**.

[54] Jiří Beran 1, Humberto Reynales 2, Airi Poder 3, Charles Y Yu 4, Punnee Pitisuttithum 5, Lee Li Yuan 6, Wim Vermeulen 7, Carole Verhoeven 8, Brett Leav 9, Bin Zhang 9, Daphne Sawlwin 10, Esther Hamers-Heijnen 7, Jonathan Edelman 11, Igor Smolenov 9; 1Vaccination and Travel Medicine Centre, Hradec Králové Czech Republic, Institute for Postgraduate Medical Education, Prague, Czech Republic.; 2Centro de Atención e Investigación Médica - CAIMED, Bogotá, Colombia.; 3Clinical Research Center, Tartu, Estonia.; 4De La Salle Health Sciences Institute, De La Salle Angelo King Medical Research Center, Dasmariñas, Cavite, Phillippines.; 5Mahidol University, Faculty of Tropical Medcine, Bangkok, Thailand.; 6Clinical Research Centre, Seri Manjung, Malaysia.; 7Seqirus Netherlands, Amsterdam, Netherlands.; 8Seqirus Netherlands, Amsterdam, Netherlands. Electronic address: carole.verhoeven@seqirus.com.; 9Seqirus, Cambridge, MA, USA.; et al. Prevention of Influenza during Mismatched Seasons in Older Adults with an MF59-Adjuvanted Quadrivalent Influenza Vaccine: A Randomised, Controlled, Multicentre, Phase 3 Efficacy Study. **2021**.

[55] Han, R.; Gomez, J. A.; de Veras, B.; Pinto, T.; Guzman-Holst, A.; Nieto, J.; van Oorschot, D. A. M. How Large Could the Public Health Impact of Introducing Recombinant Zoster Vaccination for People Aged ≥50 Years in Five Latin American Countries Be? *Hum. Vaccines Immunother.*, **2023**, 2164144. https://doi.org/10.1080/21645515.2022.2164144.

[56] Edoka, I.; Kohli-Lynch, C.; Fraser, H.; Hofman, K.; Tempia, S.; McMorrow, M.; Ramkrishna, W.; Lambach, P.; Hutubessy, R.; Cohen, C. A Cost-Effectiveness Analysis of South Africa's Seasonal Influenza Vaccination Programme. *Vaccine*, **2021**, *39* (2), 412–422. https://doi.org/10.1016/j.vaccine.2020.11.028.

[57] McMorrow, M. L.; Tempia, S.; Walaza, S.; Treurnicht, F. K.; Ramkrishna, W.; Azziz-Baumgartner, E.; Madhi, S. A.; Cohen, C. Prioritization of Risk Groups for Influenza Vaccination in Resource Limited Settings – A Case Study from South Africa. *Vaccine*, **2019**, *37* (1), 25–33. https://doi.org/10.1016/j.vaccine.2018.11.048.
[58] Feldman, C.; Dlamini, S. K.; Madhi, S. A.; Meiring, S.; von Gottberg, A.; de Beer, J. C.; de Necker, M.; Stander, M. P. The Cost-Effectiveness of Using Pneumococcal Conjugate Vaccine (PCV13) versus Pneumococcal Polysaccharide Vaccine (PPSV23), in South African Adults. *PLoS ONE*, **2020**, *15* (1), e0227945. https://doi.org/10.1371/journal.pone.0227945.
[59] Dabestani, N. M.; Leidner, A. J.; Seiber, E. E.; Kim, H.; Graitcer, S. B.; Foppa, I. M.; Bridges, C. B. A Review of the Cost-Effectiveness of Adult Influenza Vaccination and Other Preventive Services. *Prev. Med.*, **2019**, *126*, 105734. https://doi.org/10.1016/j.ypmed.2019.05.022.
[60] France, G.; Wateska, A. R.; Nowalk, M. P.; DePasse, J.; Raviotta, J. M.; Shim, E.; Zimmerman, R. K.; Smith, K. J. Potential Cost-Effectiveness of a Universal Influenza Vaccine in Older Adults. *Innov. Aging*, **2018**, *2* (3), igy035. https://doi.org/10.1093/geroni/igy035.
[61] DePasse, J. V.; Smith, K. J.; Raviotta, J. M.; Shim, E.; Nowalk, M. P.; Zimmerman, R. K.; Brown, S. T. Does Choice of Influenza Vaccine Type Change Disease Burden and Cost-Effectiveness in the United States? An Agent-Based Modeling Study. *Am. J. Epidemiol.*, **2017**, *185* (9), 822–831. https://doi.org/10.1093/aje/kww229.
[62] Chaiken, S. R.; Hersh, A. R.; Zimmermann, M. S.; Ameel, B. M.; Layoun, V. R.; Caughey, A. B. Cost-Effectiveness of Influenza Vaccination during Pregnancy. *J. Matern. Fetal Neonatal Med.*, **2022**, *35* (25), 5244–5252. https://doi.org/10.1080/14767058.2021.1876654.
[63] Manjusha Gaglani,1 Anupama Vasudevan,1 Chandni Raiyani,1 Kempapura Murthy,1 Wencong Chen,1 Michael Reis,1 Edward A Belongia,2 Huong Q McLean,2 Michael L Jackson,3 Lisa A Jackson,3 Richard K Zimmerman,4 Mary Patricia Nowalk,4 Arnold S Monto,5 Emily T Martin,5 Jessie R Chung,6 Sarah Spencer,6 Alicia M Fry,6 and Brendan Flannery6. Effectiveness of Trivalent and Quadrivalent Inactivated Vaccines Against Influenza B in the United States, 2011–2012 to 2016–2017. **2021**.
[64] Raviotta, J. M.; Smith, K. J.; DePasse, J.; Brown, S. T.; Shim, E.; Nowalk, M. P.; Wateska, A.; France, G. S.; Zimmerman, R. K. Cost-Effectiveness and Public Health Impact of Alternative Influenza Vaccination Strategies in High-Risk Adults. *Vaccine*, **2017**, *35* (42), 5708–5713. https://doi.org/10.1016/j.vaccine.2017.07.069.
[65] Ishigami, J.; Padula, W. V.; Grams, M. E.; Chang, A. R.; Jaar, B.; Gansevoort, R. T.; Bridges, J. F. P.; Kovesdy, C. P.; Uchida, S.; Coresh, J.; et al. Cost-Effectiveness of Pneumococcal Vaccination Among Patients With CKD in the United States. *Am. J. Kidney Dis.*, **2019**, *74* (1), 23–35. https://doi.org/10.1053/j.ajkd.2019.01.025.
[66] Smith, K. J.; Wateska, A. R.; Nowalk, M. P.; Lin, C. J.; Harrison, L. H.; Schaffner, W.; Zimmerman, R. K. Higher-Valency Pneumococcal Conjugate Vaccines: An Exploratory Cost-Effectiveness Analysis in U.S. Seniors. *Am. J. Prev. Med.*, **2021**, *61* (1), 28–36. https://doi.org/10.1016/j.amepre.2021.01.023.
[67] Miwako Kobayashi 1, Charles Stoecker 2, Wei Xing 3, Bo-Hyun Cho 4, Tamara Pilishvili 1. Cost-Effectiveness of Implementing 13-Valent Pneumococcal Conjugate Vaccine for U.S. Adults Aged 19 Years and Older with Underlying Conditions. **2021**.
[68] Wateska, A. R.; Nowalk, M. P.; Lin, C. J.; Harrison, L. H.; Schaffner, W.; Zimmerman, R. K.; Smith, K. J. Cost-Effectiveness of Pneumococcal Vaccination Policies and Uptake Programs in US Older Populations. *J. Am. Geriatr. Soc.*, **2020**, *68* (6), 1271. https://doi.org/10.1111/jgs.16373.
[69] Wateska, A. R.; Nowalk, M. P.; Lin, C. J.; Harrison, L. H.; Schaffner, W.; Zimmerman, R. K.; Smith, K. J. Cost-Effectiveness of Adult Pneumococcal Vaccination Policies in Underserved Minorities Aged 50–64 Years

Compared to the US General Population. *Vaccine*, **2019**, *37* (14), 2026–2033. https://doi.org/10.1016/j.vaccine.2019.01.002.

[70] Wateska, A. R.; Nowalk, M. P.; Lin, C. J.; Harrison, L. H.; Schaffner, W.; Zimmerman, R. K.; Smith, K. J. Cost-Effectiveness of Pneumococcal Vaccination and Uptake Improvement Programs in Underserved and General Population Adults Aged < 65 Years. *J. Community Health*, **2020**, *45* (1), 111–120. https://doi.org/10.1007/s10900-019-00716-8.

[71] Curran, D.; Patterson, B. J.; Van Oorschot, D.; Buck, P. O.; Carrico, J.; Hicks, K. A.; Lee, B.; Yawn, B. P. Cost-Effectiveness of an Adjuvanted Recombinant Zoster Vaccine in Older Adults in the United States Who Have Been Previously Vaccinated with Zoster Vaccine Live. *Hum. Vaccines Immunother.*, **2019**, *15* (4), 765–771. https://doi.org/10.1080/21645515.2018.1558689.

[72] Curran, D.; Patterson, B.; Varghese, L.; Van Oorschot, D.; Buck, P.; Carrico, J.; Hicks, K.; Lee, B.; Yawn, B. Cost-Effectiveness of an Adjuvanted Recombinant Zoster Vaccine in Older Adults in the United States. *Vaccine*, **2018**, *36* (33), 5037–5045. https://doi.org/10.1016/j.vaccine.2018.07.005.

[73] Meredith, N. R.; Armstrong, E. P. Cost-Effectiveness of Herpes Zoster Vaccines in the U.S.: A Systematic Review. *Prev. Med. Rep.*, **2022**, *29*, 101923. https://doi.org/10.1016/j.pmedr.2022.101923.

[74] Carpenter, C. F.; Aljassem, A.; Stassinopoulos, J.; Pisacreta, G.; Hutton, D. A Cost-Effectiveness Analysis of an Adjuvanted Subunit Vaccine for the Prevention of Herpes Zoster and Post-Herpetic Neuralgia. *Open Forum Infect. Dis.*, **2019**, *6* (7), ofz219. https://doi.org/10.1093/ofid/ofz219.

[75] Le, P.; Rothberg, M. B. Cost Effectiveness of a Shingles Vaccine Booster for Currently Vaccinated Adults in the U.S. *Am. J. Prev. Med.*, **2017**, *53* (6), 829–836. https://doi.org/10.1016/j.amepre.2017.08.029.

[76] Lee, J. K. H.; Lam, G. K. L.; Shin, T.; Kim, J.; Krishnan, A.; Greenberg, D. P.; Chit, A. Efficacy and Effectiveness of High-Dose versus Standard-Dose Influenza Vaccination for Older Adults: A Systematic Review and Meta-Analysis. *Expert Rev. Vaccines*, **2018**, *17* (5), 435–443. https://doi.org/10.1080/14760584.2018.1471989.

[77] Anne M. Butler, PhD1,2, J. Bradley Layton, PhD3, Vikas R. Dharnidharka, MD, MPH4, John; M. Sahrmann, MA1, Marissa J. Seamans, PhD5, David J. Weber, MD, MPH6, Leah J.; McGrath, PhD7. Comparative Effectiveness of High-Dose Versus Standard-Dose Influenza Vaccine Among Patients Treated by Maintenance Hemodialysis. **2020**.

[78] Katia J. Bruxvoort a, Yi Luo a, Bradley Ackerson a, Hilary C. Tanenbaum a, Lina S. Sy a, Ashesh Gandhi b, Hung Fu Tseng a. Comparison of Vaccine Effectiveness against Influenza Hospitalization of Cell-Based and Egg-Based Influenza Vaccines, 2017–2018. **2019**.

[79] Stefan Gravenstein 1, H Edward Davidson 2, Monica Taljaard 3, Jessica Ogarek 4, Pedro Gozalo 5, Lisa Han 2, Vincent Mor 6. Comparative Effectiveness of High-Dose versus Standard-Dose Influenza Vaccination on Numbers of US Nursing Home Residents Admitted to Hospital: A Cluster-Randomised Trial. **2020**.

[80] Hector S. Izurieta,1,4; Yoganand Chillarige,2; Jeffrey Kelman,3; Yuqin Wei,2; Yun Lu,1; Wenjie Xu,2; Michael Lu,2; Douglas Pratt,1; Steve Chu,3; Michael Wernecke,2; et al. Relative Effectiveness of Cell-Cultured and Egg-Based Influenza Vaccines Among Elderly Persons in the United States, 2017–2018. **2019**.

[81] Hector S. Izurieta,1; Yoganand Chillarige,2; Jeffrey Kelman,3; Yuqin Wei,2; Yun Lu,1; Wenjie Xu,2; Michael Lu,2; Douglas Pratt,1; Michael Wernecke,2; Thomas MaCurdy,2,4; et al. Relative Effectiveness of Influenza Vaccines Among the United States Elderly, 2018–2019. **2020**.

[82] Hector S Izurieta 1, Xiyuan Wu 2, Richard Forshee 1, Yun Lu 1, Heng-Ming Sung 2, Paula Ehrlich Agger 1, Yoganand Chillarige 2, Ruth Link-Gelles 3, Bradley Lufkin 2, Michael Wernecke 2, Thomas E MaCurdy 2 4, Jeffrey

Kelman 4, Kathleen Dooling 3 5. Recombinant Zoster Vaccine (Shingrix): Real-World Effectiveness in the First 2 Years Post-Licensure. **2021**.

[83] John M McLaughlin 1, Qin Jiang 1, Raul E Isturiz 1, Heather L Sings 1, David L Swerdlow 1, Bradford D Gessner 1, Ruth M Carrico 2, Paula Peyrani 2, Timothy L Wiemken 3, William A Mattingly 2, Julio A Ramirez 2, Luis Jodar 1. Effectiveness of 13-Valent Pneumococcal Conjugate Vaccine Against Hospitalization for Community-Acquired Pneumonia in Older US Adults: A Test-Negative Design. **2018**.

[84] Wateska, A. R.; Nowalk, M. P.; Lin, C. J.; Harrison, L. H.; Schaffner, W.; Zimmerman, R. K.; Smith, K. J. Pneumococcal Vaccination in Adults Aged ≥65 Years: Cost Effectiveness and Health Impact in U.S. Populations. *Am. J. Prev. Med.*, **2020**, *58* (4), 487–495. https://doi.org/10.1016/j.amepre.2019.10.022.

[85] Van Effelterre, T.; Hens, N.; White, L. J.; Gravenstein, S.; Bastian, A. R.; Buyukkaramikli, N.; Cheng, C.-Y.; Hartnett, J.; Krishnarajah, G.; Weber, K.; et al. Modeling Respiratory Syncytial Virus Adult Vaccination in the United States with a Dynamic Transmission Model. *Clin. Infect. Dis.*, **2023**, ciad161. https://doi.org/10.1093/cid/ciad161.

[86] Herring, W. L.; Zhang, Y.; Shinde, V.; Stoddard, J.; Talbird, S. E.; Rosen, B. Clinical and Economic Outcomes Associated with Respiratory Syncytial Virus Vaccination in Older Adults in the United States. *Vaccine*, **2022**, *40* (3), 483–493. https://doi.org/10.1016/j.vaccine.2021.12.002.

[87] Orly Vardeny 1, KyungMann Kim 2, Jacob A Udell 3, Jacob Joseph 4 5, Akshay S Desai 5, Michael E Farkouh 6, Sheila M Hegde 5, Adrian F Hernandez 7, Allison McGeer 8, H Keipp Talbot 9, Inder Anand 1, Deepak L Bhatt 5, Christopher P Cannon 5, David DeMets 2, J Michael Gaziano 4 5, Shaun G Goodman 10, Kristin Nichol 1, Matthew C Tattersall 11, Jonathan L Temte 12, Janet Wittes 13, Clyde Yancy 14, Brian Claggett 15, Yi Chen 2, Lu Mao 2, Thomas C Havighurst 2, Lawton S Cooper 16, Scott D Solomon 5; INVESTED Committees and Investigators. Effect of High-Dose Trivalent vs Standard-Dose Quadrivalent Influenza Vaccine on Mortality or Cardiopulmonary Hospitalization in Patients With High-Risk Cardiovascular Disease: A Randomized Clinical Trial. **2021**.

[88] Colrat, F.; Thommes, E.; Largeron, N.; Alvarez, F. P. Economic Evaluation of High-Dose Inactivated Influenza Vaccine in Adults Aged ≥65 Years: A Systematic Literature Review. *Vaccine*, **2021**, *39*, A42–A50. https://doi.org/10.1016/j.vaccine.2020.12.036.

[89] Walsh, E. E.; Pérez Marc, G.; Zareba, A. M.; Falsey, A. R.; Jiang, Q.; Patton, M.; Polack, F. P.; Llapur, C.; Doreski, P. A.; Ilangovan, K.; et al. Efficacy and Safety of a Bivalent RSV Prefusion F Vaccine in Older Adults. *N. Engl. J. Med.*, **2023**, *388* (16), 1465–1477. https://doi.org/10.1056/NEJMoa2213836.

[90] Elie A. Saade a b, Yasin Abul c d, Kevin McConeghy c d, H Edward Davidson f, Lisa Han f, Nina Joyce c, David H. Canaday b g, Leon Hsueh e, Elliott Bosco c, Stefan Gravenstein. High-Dose Influenza Vaccines for the Prevention of Hospitalization Due to Cardiovascular Events in Older Adults in the Nursing Home: Post-Hoc Analysis of a Cluster-Randomized Trial. **2022**.

[91] Kenneth E. Schmader, MD,corresponding author1,2 Christine K. Liu, MD, MSc,3,4 Theresa Harrington, MD, MPH&TM,5 Wes Rountree, MPH,6 Heidi Auerbach, MD,7 Emmanuel B. Walter, MD, MPH,6,8 Elizabeth D. Barnett, MD,9 Elizabeth P. Schlaudecker, MD, MPH,10 Chris A. Todd, MPH,6 Marek Poniewierski, MD, MS,6 Mary A. Staat, MD, MPH,10 Patricia Wodi, MD,5 and Karen R. Broder, MD5. Safety, Reactogenicity, and Health-Related Quality of Life After Trivalent Adjuvanted vs Trivalent High-Dose Inactivated Influenza Vaccines in Older Adults. **2021**.

[92] Mei Shanga,b, Jessie R Chungb, Michael L. Jacksonc; , Lisa A. Jacksonc; , Arnold S. Montod,; Emily T. Martind, Edward A. Belongiae, Huong Q. McLeane, Manjusha Gaglanif; , Kempapura; Murthyf; , Richard K. Zimmermang, Mary Patricia Nowalkg, Alicia M. Fryb, and Brendan; Flanneryb. Influenza Vaccine Effectiveness among Patients with High-Risk Medical Conditions in the United States, 2012-2016. **2018**.

[93] David K. Shay,; Yoganand Chillarige,2; Jeffrey Kelman,3; Richard A. Forshee,4; Ivo M. Foppa,1,5; Michael Wernecke,2; Yun Lu,4; Jill M. Ferdinands,1; Arjun Iyengar,; 2; et al. Comparative Effectiveness of High-Dose Versus Standard-Dose Influenza Vaccines Among US Medicare Beneficiaries in Preventing Postinfluenza Deaths During 2012–2013 and 2013–2014. **2017**.

[94] Smith, K. J.; Zimmerman, R. K.; Nowalk, M. P.; Lin, C. J. Cost Effectiveness of the 4 Pillars™ Practice Transformation Program to Improve Vaccination of Adults Aged ≥65 Years. *J. Am. Geriatr. Soc.*, **2017**, *65* (4), 763–768. https://doi.org/10.1111/jgs.14588.

[95] Navarro-Torné, A.; Montuori, E. A.; Kossyvaki, V.; Méndez, C. Burden of Pneumococcal Disease among Adults in Southern Europe (Spain, Portugal, Italy, and Greece): A Systematic Review and Meta-Analysis. *Hum. Vaccines Immunother.*, **2021**, *17* (10), 3670–3686. https://doi.org/10.1080/21645515.2021.1923348.
